# Cdk7 mediates RPB1-driven mRNA synthesis in *Toxoplasma gondii*

**DOI:** 10.1038/srep35288

**Published:** 2016-10-19

**Authors:** Abhijit S. Deshmukh, Pallabi Mitra, Mulaka Maruthi

**Affiliations:** 1 National Institute of Animal Biotechnology, Hyderabad, India; 2 International Centre for Genetic Engineering and Biotechnology, New Delhi, India; 3 Department of Animal Biology, School of Life Sciences, University of Hyderabad, Hyderabad, India

## Abstract

Cyclin-dependent kinase 7 in conjunction with CyclinH and Mat1 activates cell cycle CDKs and is a part of the general transcription factor TFIIH. Role of Cdk7 is well characterized in model eukaryotes however its relevance in protozoan parasites has not been investigated. This important regulator of key processes warrants closer examination particularly in this parasite given its unique cell cycle progression and flexible mode of replication. We report functional characterization of TgCdk7 and its partners TgCyclinH and TgMat1. Recombinant Cdk7 displays kinase activity upon binding its cyclin partner and this activity is further enhanced in presence of Mat1. The activated kinase phosphorylates C-terminal domain of TgRPB1 suggesting its role in parasite transcription. Therefore, the function of Cdk7 in CTD phosphorylation and RPB1 mediated transcription was investigated using Cdk7 inhibitor. Unphosphorylated CTD binds promoter DNA while phosphorylation by Cdk7 triggers its dissociation from DNA with implications for transcription initiation. Inhibition of Cdk7 in the parasite led to strong reduction in Serine 5 phosphorylation of TgRPB1-CTD at the promoters of constitutively expressed *actin1* and *sag1* genes with concomitant reduction of both nascent RNA synthesis and 5′-capped transcripts. Therefore, we provide compelling evidence for crucial role of TgCdk7 kinase activity in mRNA synthesis.

*Toxoplasma gondii* is an obligate intracellular protozoan parasite with a wide host range responsible for severe disease in immunocompromised individuals. The parasite displays an unique cell division cycle along with the absence of readily identifiable key controls and checkpoints^1^. The complex life cycle of this parasite comprises of alternating sexual and asexual stages in different hosts. The necessity to successfully propagate in varied host environments requires a tight regulation of gene expression. In Apicomplexa, genome surveys suggest a general conservation of basal eukaryotic transcriptional machinery^2^, however the functional identity of proteins involved as well as the mechanisms underlying the regulation of transcription have not been addressed. To fully understand and appreciate the biology of these parasites, it is important to identify and establish the key regulators of this fundamental process which currently poses a substantial knowledge gap.

The process of mRNA synthesis by the transcription machinery is a complex multi-step event which comprises of pre-initiation, initiation, promoter clearance, elongation and termination. Maturation of the synthesized nascent RNA requires further enzymatic processing which include capping, splicing, polyadenylation and cleavage that occur co-transcriptionally^3^. All of these processes are coordinated by several proteins which form dynamic complexes interacting with DNA and pre-mRNAs^3^. Phosphorylation plays a key role in mechanistic regulation of these complexes. Several protein kinases have been identified that are capable of phosphorylating proteins involved in mRNA production. One of them, cyclin dependent kinases (CDKs), represents a family of serine/threonine protein kinases that become active upon binding of a cyclin regulatory partner^4^. CDK/cyclin complexes initially identified as crucial regulators of cell cycle progression^5^; have also been implicated in transcription and mRNA processing^6^.

To attain full activity, CDKs require cyclin-binding and phosphorylation within the activation segment (T-loop)^4^. The CDK activation is primarily accomplished by a master regulatory complex which itself comprises of a CDK family member Cdk7 as its key catalytic component, called as Cyclin-dependent kinase (CDK) Activating Kinase (CAK)^7^,^8^. In addition to the catalytic subunit Cdk7, the mammalian CAK consists of a regulatory subunit CyclinH and an assembly factor MAT1 (ménage a trios1)^3^,^4^,^5^. Assembly of Cdk7-CyclinH dimeric complex instigates kinase activity which is further augmented in presence of Mat1. In this respect, phosphorylation at a conserved threonine (Thr170) residue in its own T-loop has been deemed important for Cdk7 to form a stable complex with cyclinH^7^,^8^. The ring finger protein Mat1 aids in bypassing the requirement for T loop phosphorylation in the path of steady Cdk7-CyclinH complex formation^9^,^10^,^11^,^12^,^13^. A functional CAK enzymatic complex thus formed goes on to activate several substrates including CDK enzymes important for the proper progression of the cell cycle.

Besides its role in cell cycle regulation, Cdk7-CyclinH-Mat1 complex is an essential component of general transcription factor TFIIH^14^,^15^, necessary for initiation of transcription of RNA polymerase II (Pol II)-directed genes. As part of the TFIIH, Cdk7 phosphorylates the carboxyl-terminal domain (CTD) of the largest subunit of RNA polymerase II^7^ and facilitates progression from initiation to elongation during transcription.

The CTD consists of multiple repeats of an evolutionary conserved heptapeptide with a consensus sequence Tyr_1_-Ser_2_-Pro_3_-Thr_4_-Ser_5_-Pro_6_-Ser_7_ (Y_1_S_2_P_3_T_4_S_5_P_6_S_7_). The number of repeats varies among different organisms and generally observed to increase with increasing complexity of the organisms, ranging from 26–27 in yeast to 52 in mammals^16^. The CTD phosphorylation on serine residues at positions 2 and 5 is by and large conserved and is required for the coordination of transcription with mRNA maturation^16^. Ser5 phosphorylation by Cdk7 normally occurs early in the transcription cycle coinciding with initiation^17^ while Ser2 phosphorylation by Cdk9 predominates during elongation and termination^18^,^19^. Phosphorylation at these residues has also been found to aid co-transcriptional processing of nascent RNA.

Subject to its phosphorylation state, the CTD can discriminate among its binding partners^20^,^21^. The unphosphorylated CTD binds proteins of the preinitiation complex (PIC) like the TATA-binding protein (TBP)^22^ and the Mediator complex^23^ and is implicated in the assemblage of the inactive transcription machinery on the promoter DNA. Phosphorylation of CTD also triggers polymerase to escape from the promoter and engage in productive transcript elongation^24^,^25^,^26^.

Several deviations from the general norm have been found as far as Cdk7 based CAK complex and their function is concerned. Higher eukaryotes use a single kinase complex, Cdk7/CyclinH/MAT1, to execute different fundamental cell functions including regulation of cell division and transcription^12^. In *S*. *cerevisiae*, the closest homologs of Cdk7, CyclinH, and Mat1 are Kin28, Ccl1 and Tfb3 respectively^27^,^28^, which associate with TFIIH for transcription regulating activity^26^,^29^, however, the Kin28 based complex does not display CAK activity^30^,^31^. Fission yeast *S*. *pombe* is reported to have two CAKs, the Mcs6 complex and Csk1 with both overlapping and specialized functions^32^,^33^,^34^.

CAK components have been extensively studied and explored in various model systems, however it is largely unexplored in most protozoan parasites. Only in *P. falciparum,* a homologous protein, Pfmrk which shares 46% identity with human Cdk7 has been characterized^35^. It competently phosphorylates mammalian RNAPII-CTD and histone H1 *in vitro* in presence of either human cyclin H or PfCyc-1^36^. The CTD kinase activity is reported to be stimulated in presence of PfMat1^35^. A possible role in DNA replication has been implicated, as key components of DNA replication machinery were demonstrated to be substrates of this enzyme complex in *P. falciparum*^37^. While these observations in apicomplexan indicated the importance of Cdk7 and its associated proteins in parasite lifecycle, it is imperative to understand its overall functional significance.

Given the complex life cycle and unique cell cycle progression^1^,^38^, these group of proteins merits an in depth study and analysis of their roles in *T*. *gondii*. Therefore, in the current study we systematically identify and functionally characterize Cdk7 and associated proteins CyclinH and Mat1. Importantly, we elucidate their central role in the parasite transcription whereby phosphorylation of RNA Pol II CTD at crucial serine residue by Cdk7 aids in nascent RNA synthesis and subsequent maturation by recruitment of capping enzymes.

## Results

### *In silico* identification of *T*. *gondii* Cdk7 and Mat1 ORFs

**Figure 1:**
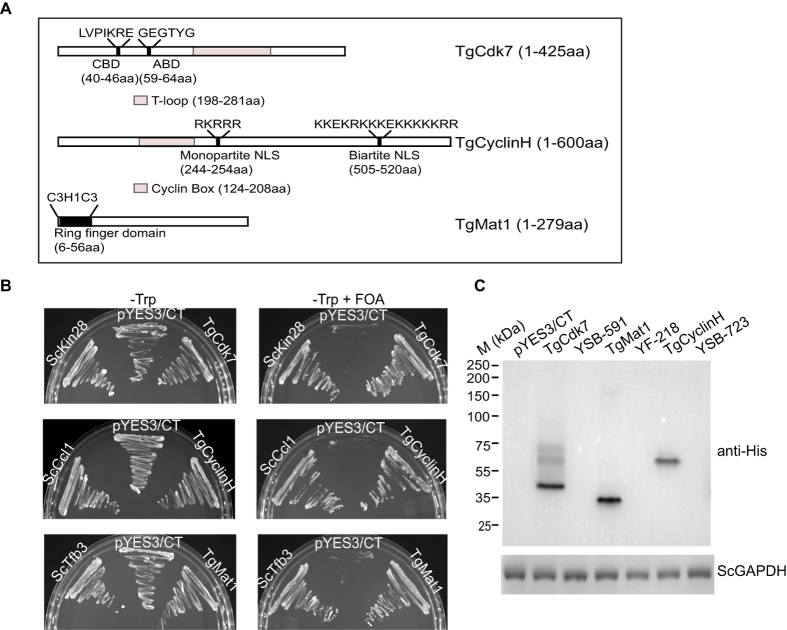
*T*. *gondii cdk7*, *cyclinH* and *mat1* genes shares functional homology with *S*. *cerevisiae kin28*, *ccl1* and *tfb3* genes respectively. (**A**) Schematic diagrams of full length *T*. *gondii* Cdk7, CyclinH and Mat1. TgCdk7: putative cyclin binding domain (CBD: 40–46 aa), ATP binding domain (ABD: 59–64 aa) and T-loop domain (198–281 aa) are shown. TgCyclinH: putative cyclin box (124–208 aa), monopartite nuclear localization signal (244–254 aa) and bipartite nuclear localization signal (505–520 aa) are shown. TgMat1: putative ring finger domain (6–56 aa) is shown. (**B**) *S*. *cerevisiae* strains with chromosomal copy deletion of *kin28*, *ccl1* and *tfb3* genes were transformed with yeast expression vector (pYES3/CT) carrying *trp* selection marker and the coding regions of respective *S*. *cerevisiae* wild type genes and *T*. *gondii cdk7*, *cyclinH* and *mat1* genes. Transformants were selected for tryptophan prototrophy by growing on medium lacking tryptophan. Selected colonies were streaked on to tryptophan-dropout medium with or without 1 mg/ml FOA. ScKin28, ScCcl1, ScTfb3 along with TgCdk7, TgCyclinH, TgMat1 could rescue the growth of *kin28*, *ccl1*, *tfb3* mutant yeast strains respectively, in the presence of FOA, whereas empty vector could not support the genetic complementation of the strains under the same experimental conditions. (**C**) *T*. *gondii* genes *cdk7*, *cyclinH* and *mat1* were expressed in the respective transformants as shown by Western blotting using ant-His antibody. *S*. *cerevisiae* GAPDH was used as a loading control (bottom panel).

We performed BLASTP searches of the *Toxoplasma gondii* genome database ( http://www.toxodb.org/) as queries of Cdk7 and Mat1 sequences from yeast, human and *Plasmodium falciparum*. This enabled us to identify TGME49_270330 and TGME49_320070, homologs of the *H*. *sapiens* Cdk7and Mat1 proteins respectively, which we call TgCdk7 and TgMat1. The predicted TgCdk7 shares 40%, 37% and 45% overall identity with the human, yeast and *P*. *falciparum* proteins respectively (Supplementary Fig. S1A). The kinase domain shares upto 47% identity with its counterparts in these organisms (Supplementary Fig. S1A). In addition, TgCdk7 possesses the conserved cyclin binding domain (40–46 aa), the ATP binding domain (59–64 aa) and the T-loop (198–281 aa) (Fig. 1A). TgCyclinH protein, previously identified through yeast two hybrid analyses^39^, shares overall ~21% identity with the human, yeast and *P*. *falciparum* proteins (Supplementary Fig. S1B). The highest homology was identified in the cyclin box (124–208 aa), a region responsible for Cdk binding. The cyclin box shares overall 26%, 21% and 34% identity with the human, yeast and *P*. *falciparum* cyclin box respectively (Supplementary Fig. S1B). Along with cyclin box, TgCyclinH also contains monopartite (244–254 aa) and bipartite (505–520 aa) nuclear localization signals, in addition to unique N- and C-terminal extensions (Fig. 1A). Similarly, alignment among Mat1 proteins revealed that the predicted TgMat1 shares 33% identity with *P*. *falciparum* and ~30% identity with yeast and human homologs (Supplementary Fig. S1C). *T*. *gondii* Mat1 protein possesses highly conserved ring finger domain (6–56 aa) at the N-terminus of the protein (Fig. 1A) which shares 39%, 36% and 42% identity with the human, yeast and *P*. *falciparum* ring finger domains respectively (Supplementary Fig. S1C). Ring-finger domain is of the Cys3HisCys4 type and has been implicated in protein-protein interaction and in directing the CDK complex to specific substrates^40^,^41^.

### *T*. *gondii cdk7*, *cyclinH* and *mat1* genes functionally complement *S*. *cerevisiae kin28*, *ccl1* and *tfb3* genes respectively

To investigate whether *Toxoplasma* Cdk7, CyclinH and Mat1 are CAK homologs, we performed a functional complementation assay. For complementation studies, yeast mutant strains with a deletion of chromosomal copy of Kin28 (YSB-591), Ccl1 (YSB-723) or Tfb3 (YF-218) and bearing the wild-type copy of the respective gene in a plasmid containing an *ura3* marker^42^ were transformed with plasmids for expressing *Sckin28*, *Scccl1* and *Sctfb3*, pYES3/CT (empty vector), or *T*. *gondii cdk7*, *cyclinH* and *mat1* genes under a galactose-inducible promoter with a tryptophan marker and C-terminal His-tag. Following transformation, the transformants were grown either in the absence or presence of 5-fluoroorotic acid (5-FOA) for the selection of viable yeast cells in minimal medium lacking tryptophan with or without FOA. The results revealed that mutant cells expressing TgCdk7, TgCyclinH or TgMat1 were proficient for growth on SD-Trp+FOA much like the situation with self-complemented strain, whereas those mutant cells transformed with pYES3/CT empty vector failed to grow on SD-Trp+FOA (Fig. 1B). This result clearly showed that TgCdk7, TgCyclinH, and TgMat1 were capable of fully functionally complementing the function of Kin28, Ccl1 and Tfb3 respectively within the yeast mutant strains. All three *Toxoplasma* genes Cdk7 (~46 kDa), CyclinH (~66 kDa) and Mat1 (~32 kDa) were expressed in the transformed yeast but not in the untransformed yeast mutant strains (Fig. 1C). The expression of control gene *Scgapdh* (glyceraldehyde-3-phosphate dehydrogenase) was found to be similar in all the transformed or untransformed yeast strains. The results establish that the TgCAK related proteins Cdk7, CyclinH and Mat1 are true functional counterparts of ScKin28, ScCcl1 and ScTfb3 respectively.

### *T*. *gondii cdk7*, *cyclinH* and *mat1* genes express in the parasite

**Figure 2:**
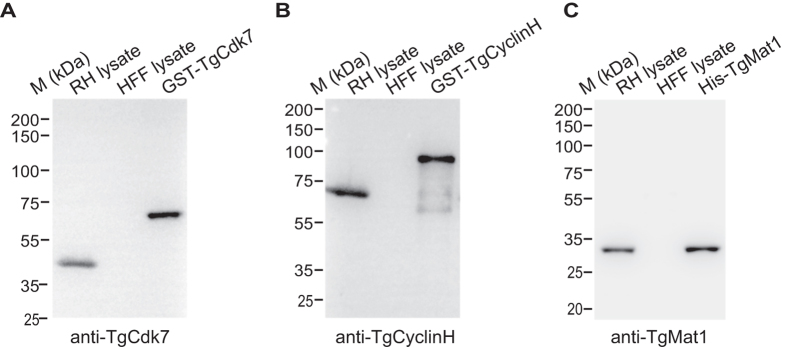
Generation of polyclonal antibodies against TgCdk7, TgCyclinH and TgMat1 which specifically identifies the native protein in the parasite. (**A**) Polyclonal antibodies raised against TgCdk7 were specific as western blot analysis with immune sera recognized the band of expected size (~46 kDa) in the native parasite lysate. The antibodies also recognized GST-TgCdk7 recombinant protein (~72 kDa). (**B**) Similarly polyclonal antibodies recognized TgCyclinH native (~65 kDa) and recombinant (GST-tag) proteins (~92 kDa). (**C**) TgMat1 antibody identified both native and recombinant (His-tag) proteins at the predicted size (~32 kDa). All the three antibodies raised did not display cross-reactivity with HFF proteins.

To determine the endogenous expression of *T*. *gondii* CAK related components in the parasites, full length Cdk7_1-425_, CyclinH_1-600_ and Mat1_1-279_ were amplified from *T*. *gondii* cDNA. The amplicons for Cdk7 and CyclinH were cloned in to the pGEX-6P-2 vector, introducing an N-terminal glutathione S-transferase fusion and Mat1 amplicon into the pET-28a vector, introducing an N-terminal histidine fusion and expressed in *E*. *coli*. Both Cdk7 and CyclinH were expressed as soluble proteins and Mat1 protein was expressed as a part of inclusion bodies. Recombinant proteins were purified using affinity chromatography. Recombinant TgCdk7 and TgCyclinH showed an expected mobility of ~72 kDa and ~92 kDa respectively (including GST protein ~26 kDa) while TgMat1 migrated at ~32 kDa in SDS-PAGE analysis (Supplementary Fig. S2). Each of the recombinant purified protein was used as antigen to raise specific polyclonal antibodies in mice and rabbits. The Cdk7 antiserum recognized the recombinant protein (~72 kDa) and a band of expected size at about 46 kDa in the parasite lysate (Fig. 2A). Similarly, the TgCyclinH antiserum recognized the recombinant protein (~92 kDa) and a predicted size band at about 65 kDa in the parasite lysate (Fig. 2B) whereas Mat1 specific polyclonal antibody recognized both the recombinant protein and a band of expected size in the parasite lysate (~32 kDa) (Fig. 2C). All the three polyclonal antibodies did not show cross-reactivity with HFF proteins.

### *T*. *gondii* Cdk7, CyclinH and Mat1 display interactions

**Figure 3:**
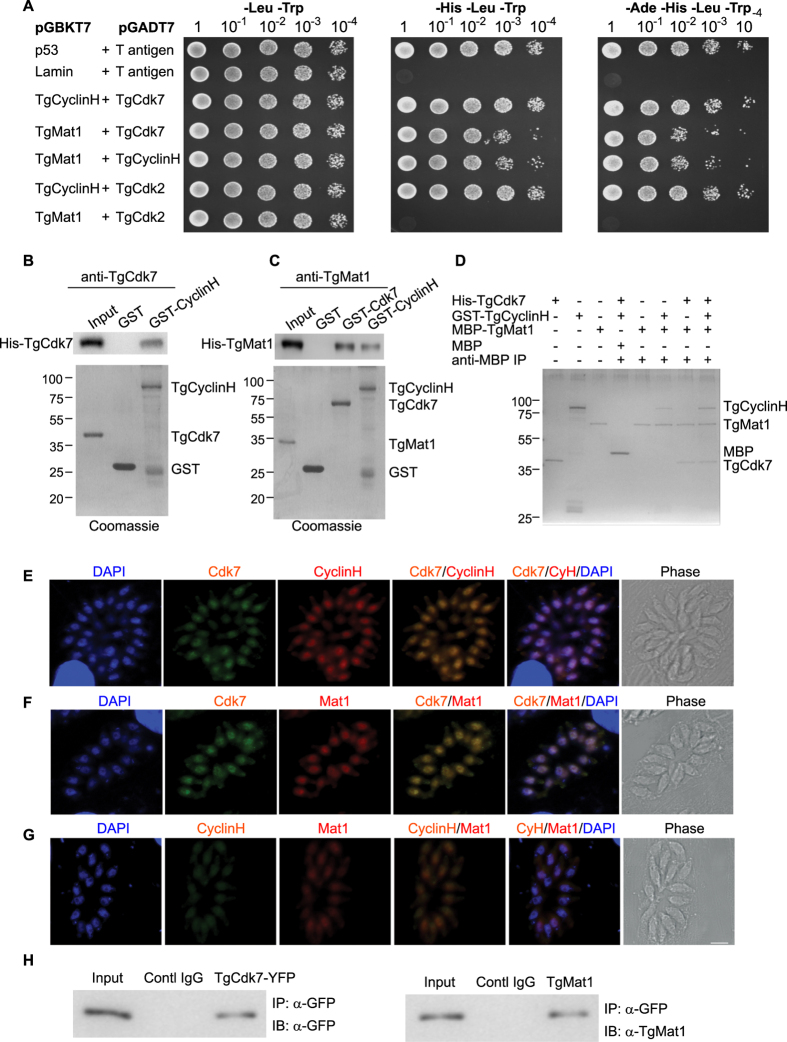
*T*. *gondii* Cdk7, CyclinH and Mat1 interact with each other. (**A**) Interactions between TgCdk7, TgCyclinH and TgMat1 were detected by yeast two hybrid analyses. *T*. *gondii* CyclinH and Mat1 proteins fused to the GAL4 DNA-binding domain (BD) were expressed in combination with either Cdk7 or CyclinH or Cdk2 fused to the GAL4 activation domain (AD) in yeast strain Y2HGold. Each transformant was spotted in serial dilutions from 10^1^ to 10^−4^ on SD plates lacking Leu and Trp (DDO); His, Leu and Trp (TDO) and Ade, His, Leu and Trp (QDO). Transformants TgCyclinH-BD/TgCdk7-AD, TgMat1-BD/TgCdk7-AD, TgMat1-BD/TgCyclinH-AD, TgCyclinH-BD/TgCdk2-AD and not the TgMat1-BD/TgCdk2-AD were capable of growth in the absence of Ade and His. The p53-BD/T antigen-AD and Lamin-BD/T antigen-AD were used as a positive and negative control interaction respectively. (**B**) GST pull down assays were performed using GST beads bound TgCyclinH and GST alone proteins in the presence of His-Cdk7. Western blot analysis using anti-Cdk7 antibody showed the specific binding of TgCyclinH with TgCdk7. The bottom panel showed the Coomassie-stained gel following protein transfer as loading control. (**C**) Similar interaction experiment using GST beads bound TgCyclinH, TgCdk7 and GST alone proteins in the presence of His-TgMat1 showed the specific interactions of TgCdk7 and TgCyclinH with TgMat1. (**D**) TgCdk7, TgCyclinH and TgMat1 proteins form a complex *in vitro*. IP with anti-MBP antibody could pull down His-TgCdk7 and GST-TgCyclinH along with MBP-TgMat1. Proteins eluted from anti-MBP antibody crosslinked agarose beads were subjected to SDS-PAGE and silver staining analysis. (**E**) Immunofluorescence assay (IFA) in RH strain *T*. *gondii*. IFA using anti-Cdk7 and anti-CyclinH antibodies showed colocalization in the parasite nucleus. (**F**) Similarly, IFA using anti-Cdk7 and anti-Mat1 antibodies showed nuclear colocalization. (**G**) IF using anti-CyclinH and anti-Mat1 antibodies showed colocalization mostly in the parasite nucleus. DAPI was used to stain the parasite nucleus. Scale bar 5 μm. (**H**) IP using anti-GFP antibody or control IgG followed by immunoblotting (IB) with anti-Mat1 antibody. TgMat1 co-immunoprecipitated with TgCdk7-YFP using specific anti-GFP antibody, but not with control IgG.

Given that the *T*. *gondii* CAK like components functionally complemented *S*. *cerevisiae* mutant strains; we wanted to explore the possibility of complex formation like their counterparts in yeast. In order to experimentally test this hypothesis in a systematic fashion, all TgCAK subunits were cloned into either pGBKT7-BD or pGADT7-AD vector and tested for their ability to interact with each other in yeast two hybrid assay. Previously demonstrated TgCdk2 (a putative Cdk1 homolog) interaction with TgCyclinH was used a positive control for *Toxoplasma* proteins^39^,^43^. The TgCdk7, TgCyclinH and TgCdk2 proteins fused to the GAL4-AD were transformed in pairwise combinations with either TgMat1 or TgCyclinH fused to the GAL4-BD into the yeast strain Y2HGold. Interaction between two given proteins was visualized by monitoring growth on medium lacking histidine. All TgCAK like protein components tested were able to interact with each other i.e. TgCdk7-TgCyclinH, TgCdk7-TgMat1 and TgCyclinH-TgMat1 (Fig. 3A). TgCdk2 protein interacts with TgCyclinH in our experimental conditions as well (Fig. 3A). The transformants of TgMat1 and TgCdk2 did not restore growth in the absence of histidine and thus do not interact with each other (Fig. 3A). Interactions between p53 with T-antigen and Lamin with T-antigen were used a positive and negative control respectively. The yeast two-hybrid analyses strongly suggest that TgCAK proteins interact with each other and possibly form a complex.

The yeast two-hybrid results were further supported by GST pull-down assay. In the GST pull-down assay (Fig. 3B), GST bound TgCyclinH (on bead) was incubated in presence of His-TgCdk7 protein; while bead bound GST protein alone was used as a control. Upon western blot analyses with TgCdk7 antiserum, we found that TgCyclinH could bind TgCdk7 with no nonspecific binding observed with GST protein. In a similar experiment GST beads bound proteins TgCyclinH and TgCdk7 were able to bind to His-TgMat1, when immunoblotted with TgMat1 antibodies (Fig. 3C), while no interaction of His-TgMat1 with GST protein was observed. Similarly, when interactions of TgCdk2-His with either GST-TgCyclinH or GST-TgMat1 protein were tested, interaction could only be detected with TgCyclinH and not with GST-Mat1 or GST protein (Supplementary Fig. S3).

Once we confirmed the pairwise interactions, we further wanted to investigate the possible *in vitro* ternary interaction between TgMat1, TgCdk7 and TgCyclinH. In order to examine the same, pull-down assays were performed as described previously^37^. MBP-TgMat1 was mixed with TgCdk7 in the presence or absence of TgCyclinH and immunoprecipitated with anti-MBP antibody crosslinked to aminoLink agarose. The immunoprecipitated proteins were then visualized with silver staining. MBP antibodies could pullout TgCdk7 as well as TgCyclinH along with TgMat1 confirming the complex formation *in vitro* (Fig. 3D). In addition, TgMat1 was found to bind independently to both TgCdk7 and TgCyclinH (Fig. 3D) consistent with the GST pull down assay. A control experiment consisting of the MBP tag alone did not pull down any of the proteins. Together, our *in vitro* data suggest that the Cdk7, CyclinH and Mat1 interact with each other and possibly form a complex.

To further confirm the pull-down results, indirect immunofluorescence experiments were performed with antibodies directed against proteins TgCdk7, TgCyclinH and TgMat1 in the replicating-tachyzoites. The results showed that each of these proteins is enriched in the parasite nucleus (Fig. 3E–G). Double-labeling experiments using either Cdk7 antibody in combination with CyclinH antibody or Mat1 antibody or CyclinH in combination with Mat1 antibody demonstrated that the three proteins colocalize in the parasite nucleus.

Following confirmation of complex formation by recombinant purified proteins TgCdk7, TgMat1 and TgCyclinH, our attempts to validate these interactions in the parasite using polyclonal antibodies were unable to capture the anticipated interactions. Taking the cue from the i*n vitro* binding studies which show that components of the complex can be co-precipitated if they are tagged at the protein termini, we ectopically expressed TgCdk7, TgCyclinH and TgMat1 proteins either as YFP or mCherry fusions, which was confirmed by western blot analysis using respective tag antibodies (Supplementary Fig. S4A–C). All three proteins mostly localized in the parasite nucleus (Supplementary Fig. S4D–F) mirroring the immunofluorescence data. Co-IP demonstrated the interaction of TgMat1 with TgCdk7-YFP in the parasite (Fig. 3H). However, the other anticipated interactions of TgCdk7 with TgCyclinH and TgMat1 with TgCyclinH could not be detected using this approach. This may be due to the transient nature of these interactions which makes it difficult to be captured by standard immunoprecipitation experiments.

### TgCdk7 displays CTD-kinase activity

**Figure 4:**
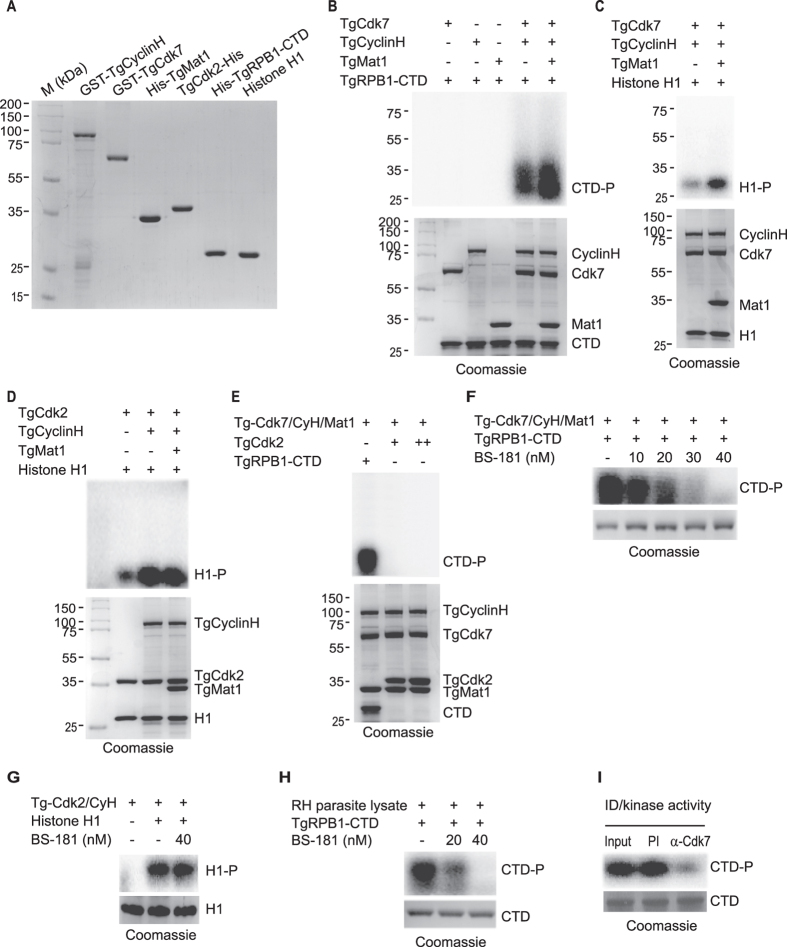
TgCdk7 is an active kinase which can phosphorylate TgRPB1-CTD. (**A**) Coomassie gel showing purified recombinant GST-TgCyclinH, GST-TgCdk7, His-TgMat1, TgCdk2-His, His-TgRPB1-CTD and histone H1 proteins used in the kinase assays. (**B**) TgRPB1-CTD was phosphorylated (lane four) in the presence of TgCdk7 and TgCyclinH. The phosphorylation of TgRPB1-CTD was increased in the presence Mat1 (lane five). TgCdk7, TgCyclinH or TgMat1 alone failed to phosphorylate TgRPB1-CTD. Coomassie stained gel (lower panel) show loading of all the proteins and equal loading of TgRPB1-CTD. (**C**) Similarly, TgCdk7/TgCyclinH phosphorylated universal kinase substrate histone H1 and the effect was enhanced in the presence of Mat1. (**D**) Kinase assays were performed using TgCdk2, TgCyclinH and TgMat1 in the presence of histone H1 as a substrate. Weak phosphorylation of histone H1 was detected with alone TgCdk2. Robust phosphorylation of histone H1was observed in the presence of activated kinase Tg-Cdk2/CyclinH. The phosphorylation level of histone H1 remained unchanged in the presence of TgMat1. Coomassie stained gel (lower panel) show equal loading of histone H1. (**E**) Tg-Cdk7/CyclinH/Mat1 did not phosphorylate TgCdk2 (lane two and three) but phosphorylate TgRPB1-CTD (lane one). (**F**) TgCdk7 kinase activity showed progressive reduction in presence of increasing concentration of BS-181 *in vitro*. The CTD kinase activity was completely abolished at 40 nM concentration. Coomassie stained gel show equal loading of TgRPB1-CTD (lower panel). (**G**) TgCdk2 kinase activity was not inhibited at 40 nM concentration of BS-181. (**H**) Kinase assay using lysate prepared from RH parasites treated with Cdk7 inhibitor, BS-181 showed diminished phosphorylation of TgRPB1-CTD. (**I**) TgCdk7 immunodepleted parasite lysate showed decline in TgRPB1-CTD phosphorylation. Pre-immune sera depleted parasite lysate was used as control. Coomassie stained gel show equal loading of TgRPB1-CTD.

Human Cdk7 associates with CyclinH for kinase activity which is significantly increased in the presence of an assembly factor Mat1^9^,^10^. To investigate whether the kinase activity of TgCdk7 requires TgCyclinH binding and the addition of a third subunit TgMat1 could influence the kinase activity of TgCdk7, *in vitro* kinase assays were performed using recombinant GST-TgCyclinH, GST-TgCdk7, His-TgMat1 and TgCdk2-His proteins to determine their ability to phosphorylate His-TgRPB1-CTD and histone H1 (Fig. 4A). Protein expression profiles of TgCdk2 and TgRPB1-CTD are shown in supplementary Fig. S5. In *T*. *gondii*, RPB1 (TGME49_225260) is the largest subunit of RNA polymerase II encoded by the TgPOLR2A gene. TgCdk7 displayed CTD kinase activity only in presence of TgCyclinH while none of the components including TgCdk7, TgCyclinH and TgMat1 individually phosphorylated TgRPB1-CTD (Fig. 4B). Interestingly, addition of TgMat1 to TgCdk7/TgCyclinH reaction substantially augmented the CTD kinase activity (Fig. 4B). We further verified these kinase complexes in *in vitro* kinase assay using universal substrate histone H1. Histone H1 also showed enhanced phosphorylation by TgCdk7/TgCyclinH in the presence of TgMat1 (Fig. 4C).

TgCdk2 has been previously demonstrated to display kinase activity in the presence of substrate histone H1^43^. In the light of the finding that TgCdk2 interacts with TgCyclinH (Fig. 3A) we wanted to determine whether addition of TgMat1 in the presence of TgCdk2 and TgCyclinH had any influence on the TgCdk2 kinase activity. To test it, we checked the levels of histone H1 phosphorylation in the presence of the activated kinase, Tg-Cdk2/CyclinH and Tg-Cdk2/CyclinH/Mat1. While TgCdk2 displayed basal kinase activity towards histone H1 even in absence of TgCyclinH, there was no appreciable change in the kinase activity of Tg-Cdk2/CyclinH towards histone H1 on addition of TgMat1 (Fig. 4D). In our experimental conditions we could not detect autophosphorylation of either TgCdk7 or TgCdk2 in the presence of TgCyclinH.

The mammalian Cdk7-CyclinH-Mat1 complex has a dual role both as CTD kinase involved in the regulation of transcription and as the CDK activating kinase (CAK) responsible for phosphorylation and activating Cdk2 kinase in the regulation of cell cycle progression^7^. To test a similar role for TgCdk7, we examined the ability of TgCdk7 to phosphorylate TgCdk2. Kinase reactions were performed using activated kinase complex Tg-Cdk7/CyclinH/Mat1 with either TgRPB1-CTD (positive control) or TgCdk2 protein as substrates, we observed that TgCdk7 could phosphorylate CTD but not TgCdk2 in our experimental conditions (Fig. 4E). These results indicate that TgCdk2 may not be a substrate for TgCdk7 and suggest that Tg-Cdk7/CyclinH/Mat1 is probably not the requisite CAK capable of activating TgCdk2. It will be interesting to investigate now whether this CAK like multiprotein complex has the ability to activate any of the wide arrays of *T*. *gondii* CDKs before making a conclusive remark on its potential CAK activity.

Chemical inhibitor BS-181 has been previously reported to effectively abrogate the mammalian Cdk7 kinase activity *in vitro* as well as *in vivo*^44^. In order to examine whether BS-181 could also inhibit the TgCdk7 kinase activity, we checked TgCdk7 kinase activity in presence of different concentrations of BS-181 *in vitro.* We observed diminished phosphorylation of TgRPB1-CTD with increasing concentration of BS-181 which is nearly abolished at ~40 nM concentration (Fig. 4F). While 40 nM concentration of BS-181 showed profound effect on TgCdk7 kinase activity but failed to inhibit TgCdk2 kinase activity (Fig. 4G) suggesting the specificity of this inhibitor to TgCdk7. These results were consistent with previous studies where similar dosage of BS-181 inhibited the respective kinase activity *in vitro* while for *in vivo* studies micromolar levels of the chemical inhibitor could competently inhibit the kinase activity^44^. Taking that cue, we first tested the effect of 5 μM and 10 μM concentrations of BS-181 (which is less than the concentrations used for inhibiting mammalian Cdk7^44^) on *T*. *gondii* proliferation in HFF cells. After 24 h of inhibitor treatment, substantial inhibition of *T*. *gondii* growth and proliferation by BS-181 at both the concentrations was observed with more pronounced effect at 10 μM concentration (Supplementary Fig. S6). In order to assess whether the observations following treatment with BS-181 is an outcome of a TgCdk7 inhibitory effect and not a possible indirect effect of BS-181 induced apoptosis of the host cell, we checked for Annexin V staining of the host HFF cells following BS-181 treatment for 24 h. We observed less than 5% of HFF cells staining positive for Annexin V with either DMSO or 5 μM or 10 μM BS-181 (Supplementary Fig. S7). These results were consistent with previous studies where similar dosages of BS-181 treatment result in less than 10% of apopototic cells^44^.

To ascertain that the TgCdk7 is indeed responsible for TgRPB1-CTD phosphorylation, we performed kinase assays using RH parasite lysate treated with two different concentrations (20 and 40 nM) of BS-181. Phosphorylation of TgRPB1-CTD was considerably reduced in the presence of 20 nM while it was completely abolished at 40 nM concentration of BS-181, indicating that TgRPB1-CTD is possibly phosphorylated by TgCdk7 in the parasite (Fig. 4H).

To further strengthen the candidature of TgCdk7 as the CTD kinase, immunodepletion (ID) was performed with TgCdk7 antibodies in RH parasite lysate followed by kinase assay using TgRPB1-CTD as the substrate. We found reduced phosphorylation of TgRPB1-CTD when incubated with TgCdk7 immunodepleted parasite lysate compared to pre-immune control (Fig. 4I).

### TgCdk7 phosphorylates TgRPB1 at crucial serine residue in the functional heptapeptide repeats

**Figure 5:**
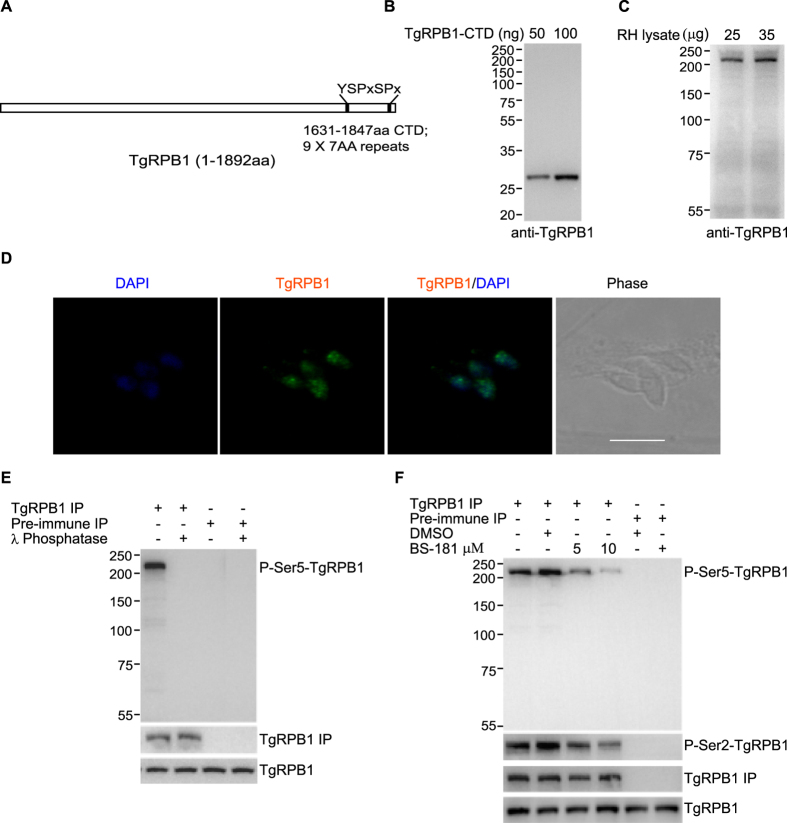
TgCdk7 phosphorylates TgRPB1-CTD at serine 5 of the heptapeptide repeats. (**A**) Schematic diagram of TgRPB1. The carboxyl-terminal domain (1631–1847 aa) containing putative nine heptad repeats of YSPxSPx is depicted. (**B,C**) Polyclonal antibody raised against recombinant TgRPB1-CTD recognized the recombinant (~27 kDa) and native proteins (~209 kDa) at the expected sizes in a concentration dependent manner. (**D**) IF analysis using anti-TgRPB1 antibody showed punctate nuclear foci in the parasite. Scale bar 5 μm. (**E**) Phosphorylation of Ser5 residue of heptapeptide repeats in TgRPB1 was demonstrated using IP with anti-TgRPB1 antibody followed by λ-phosphatase treatment and Western blot analysis using P-Ser5 antibody. No band was detected in the pre-immune IP. Similar levels of TgRPB1 in lanes 1 and 2 suggested equal pull down of TgRPB1. (**F**) Inhibition of TgCdk7 by BS-181 followed by TgRPB1 IP and western blot using P-Ser5 antibody and P-Ser2 antibody. Western blot shows abrogation of P-Ser5 while P-Ser2 is also affected. No inhibitory effect was observed for DMSO treated parasite. No band was detected in the pre-immune IP. Similar levels of TgRPB1 in lanes 1, 2, 3 and 4 suggested equal pull down of TgRPB1. Similar level of TgRPB1 (bottom panel E,F) indicates equal amount of parasite proteins used for IP.

The CTD of RNA polymerase II consists of consensus multiple heptapeptide repeats of Y_1_S_2_P_3_T_4_S_5_P_6_S_7_ and is involved in binding of several proteins^16^. This repeat region is highly phosphorylated in transcribing RNAPII. We searched for the presence of such heptapeptide repeats in the TgRPB1 sequence manually and by using bioinformatics tools, and found no consensus YSPTSPS sequence but mixture of nine putative heptapeptide YSPxSPx sequences (where x can be any amino acid) at the C-terminal domain (1631–1847 aa) (Fig. 5A). It is important to note that substitutions at position 4 and 7 in the heptapeptide repeats are reported in many organisms^16^.

To examine the role of TgRPB1-CTD in transcription we first raised the polyclonal antibodies against bacterially expressed His-TgRPB1-CTD. TgRPB1 antiserum recognized the recombinant protein (~27 kDa) as well as the native parasite protein (~209 kDa) at the desired sizes in a concentration dependent manner (Fig. 5B,C). The antibody recognized the band of expected size in the RH parasite lysate whereas no band was observed in the HFF lysate (Supplementary Fig. S8).

Indirect immunofluorescence assays were performed to define the localization of TgRPB1 in RH tachyzoites. We found that TgRPB1 is predominantly localized in the parasite nucleus and show punctate staining pattern possibly representing the active transcription sites (Fig. 5D).

In mammalian cells the CTD of RNAPII is phosphorylated at least on three serine residues (Ser-2, Ser-5 and Ser-7) in the heptapeptide sequence^16^. TgRPB1-CTD has only two conserved serine residues at 2^nd^ and 5^th^ positions in all nine identified heptapeptide sequences (Fig. 5A), indicating phosphorylation of these residues during *T*. *gondii* transcription. To examine the *in vivo* phosphorylation of endogenous TgRPB1 on Ser5 of CTD heptatpeptide, we first performed immunoprecipitation of TgRPB1 using anti-TgRPB1 antibody from the parasite lysate, and the immunoprecipitated protein was treated with λ-phosphatase followed by Western blot analysis using P-Ser5 antibody. We observed disappearance of the phosphorylated protein band (Fig. 5E), confirming that endogenous TgRPB1 is phosphorylated on Ser5 of CTD.

Further to examine the specificity of TgCdk7-mediated TgRPB1-CTD Ser5 phosphorylation in heptapeptide, we performed immunoprecipitation of TgRPB1 from parasite treated with either DMSO or Cdk7 inhibitor BS-181 followed by Western blot with P-Ser5 antibody. We observed reduced band of P-Ser5-TgRPB1 in the inhibitor treated parasites while DMSO treated control remains unaltered (Fig. 5F). Subsequent to the P-Ser5 enrichment at the promoter region, CTD Ser2 phosphorylation (P-Ser2) rises downstream of the transcription start site and coincides with the entry of RNAPII in the productive elongation phase of transcription^17^,^18^,^19^. Therefore, reduction in P-Ser5 results in subsequent reduction in P-Ser2. To test this, we checked the level of P-Ser2 upon Cdk7 inhibition and observed that P-Ser2 at TgRPB1-CTD is moderately affected most likely due to the fact that these two events are not independent of each other (Fig. 5F). The observation that reduction in CTD P-Ser5 leads to subsequent reduction in P-Ser2 in *Toxoplasma* is consistent with similar observation made previously^17^,^18^,^19^. These reports demonstrate that phosphorylation at Ser2 of CTD by Cdk9 is influenced by phosphorylation at Ser5 by Cdk7 which is prerequisite for activation of Cdk9 for Ser2 phosphorylation. These results highlighted the role of TgCdk7 in phosphorylation of CTD probably at Serine 5 residue in the heptapeptide repeats in overall transcriptional regulation in *Toxoplasma*.

### Phosphorylation of TgRPB1-CTD by TgCdk7 affects its DNA binding activity

**Figure 6:**
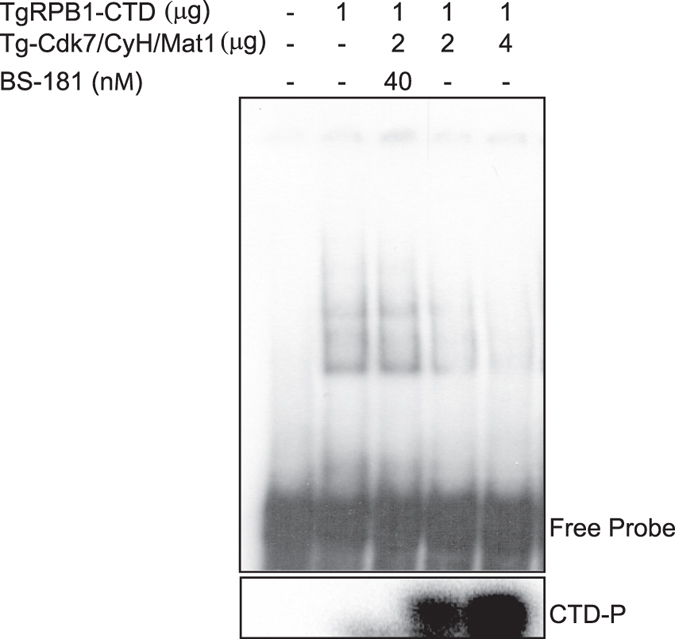
Phosphorylation of TgRPB1-CTD abrogates promoter DNA binding. Unphosphorylated CTD binds to DNA (lane 2). Binding was substantially decreased in a concentration dependent manner when CTD is phosphorylated by activated kinase Tg-Cdk7/CyclinH/Mat1. The DNA binding remained unaltered in the presence of inhibitor BS-181 (lane 3). Kinase reaction control showing CTD phosphorylation in the presence of activated kinase is shown in lower panel.

The mammalian CTD of RNA polymerase II in its unphosphorylated form binds to promoter DNA which is (CTD/DNA complex) then recognized and phosphorylated by Cdk7^21^. The phosphorylated CTD is then dissociated from DNA and allow transcription initiation^21^. To examine whether TgCdk7 mediated CTD phosphorylation also influences CTD binding to the promoter DNA, we tested the TgTubulin1 gene (TgTUB1) promoter DNA (Supplementary Fig. S9) binding activity of unphosphorylated, and phosphorylated TgRPB1-CTD in presence and absence of BS-181. The unphosphorylated CTD was found to bind promoter DNA and upon phosphorylation by activated kinase Tg-Cdk7/CyclinH/Mat1 CTD showed reduction in DNA binding in a concentration dependent manner (Fig. 6) whereas in the presence of inhibitor BS-181, the DNA binding activity of CTD remained unchanged (Fig. 6, lane 3). Therefore, our data demonstrated that unphosphorylated TgRPB1-CTD is able to bind promoter DNA and this CTD/DNA complex is probably required in the context of the pre-initiation complex indicating the conserved mechanism of transcription initiation.

### Inhibition of TgCdk7 causes reduction in *de novo* mRNA synthesis

**Figure 7:**
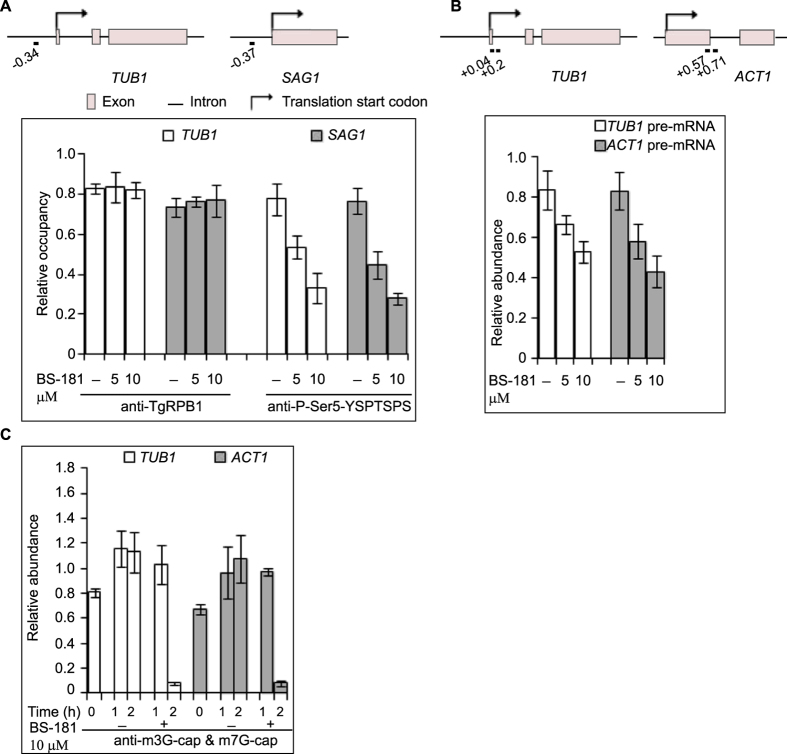
TgCdk7 inhibition affects *de novo* transcription by TgRPB1. (**A**) Schematic diagram showing approximate positions in Kb of the ChIP-qPCR products relative to the *TUB1* and *SAG1* start codons (upper panel). Occupancy of unphosphorylated TgRPB1 and P-Ser5 at the *TUB1* and *SAG1* promoters was determined using ChIP analysis. Immunoprecipitated DNA from inhibitor treated parasites was quantified and normalized by calculating fold enrichment over nontranscribed telomere DNA^62^, followed by real-time PCR. Prominent reduction of Ser5 phosphorylation was observed on *TUB1* and *ACT1* promoters at both the concentrations of inhibitor used with nearly two fold reductions at 10 μM of BS-181. However, unphosphorylated TgRPB1 occupancy at these promoters remains unchanged. (**B**) Schematic diagram showing approximate locations in Kb of the forward and reverse primers of qRT-PCR products relative to the *TUB1* and *ACT1* start codons (upper panel). Quantitative RT-PCR of the pre-mRNA levels of *TUB1* and *ACT1* was undertaken. A primer set targeting a region of *ACT1* corresponding to exon1 and 2 was used as the control. Noticeable reduction was observed in the pre-mRNA levels of both the genes at 5 μM and 10 μM of BS-181. (**C**) Marked reduction of capped transcripts following TgCdk7 inhibition was observed. Parasites were harvested at time 0 and at 1 and 2 h after treatment with 10 μM of BS-181. RNA-IP with anti-cap (m3G-cap & m7G-cap) antibody followed by quantitative RT-PCR using *TUB1* and *ACT1*-specific primers showed marked reduction in 5′-capping of *TUB1* and *ACT1* transcripts after 2 h of inhibitor treatment.

We observed that BS-181 inhibit TgCdk7 kinase activity *in vitro* (Fig. 4F) and decrease Ser5-CTD phosphorylation *in vivo* (Fig. 5E,F); however, to determine the effect of inhibitor on association of phosphorylated Ser-5-CTD with well characterized active promoters^45^,^46^, we examined the levels of Ser-5-CTD phosphorylation at *TgTUB1* and *TgSAG1* promoters by ChIP using P-Ser5 antibody in the presence and absence of inhibitor treatment. We observed that Ser-5 phosphorylation was considerably diminished on both *TUB1* and *SAG1* promoters following inhibitor treatment for 2 h (Fig. 7A) while general occupancy of these promoters by unphosphorylated TgRPB1 remains unaltered as assessed by ChIP using TgRPB1 antibody.

To examine the role of TgCdk7 in TgRPB1-driven transcription, we checked the effect of the inhibitor on formation of nascent RNA at endogenous parasite genes. Parasites were treated with 5 μM and 10 μM concentrations of BS-181 for 2 h and were then harvested for subsequent RNA purification and cDNA preparation. Because of the rapid action of the splicing machinery in comparison to the relatively long half-lives of mature mRNAs, primers targeting specific exon-intron junctions were used for quantitative analysis of newly synthesized pre-mRNAs^47^. The nascent RNA levels (determined using primers targeting exon-intron junction) of two highly expressed *Toxoplasma* genes i.e. *TUB1* and *ACT1* (Actin) were measured by qRT-PCR. Reduction in nascent RNA was found following Cdk7 inhibition (Fig. 7B). These results are consistent with data observed for human cancer cell lines^47^,^48^.

Ser5 phosphorylation occurs at initiation and is critical for recruitment and activation of capping enzymes^49^. To determine the effect of inhibition of CTD phosphorylation on capping enzyme bound nascent transcript, we performed ChIP-qRT-PCR assay with anti-m3G-cap & m7G-cap antibody in the parasites treated with 10 μM BS-181 up to 2 h. Parasites were harvested at 0, 1 and 2 h post inhibitor treatment followed by RNA-ChIP analysis. Quantitative RT-PCR was performed using *TUB1* and *ACT1* specific primers on the immunoprecipitated RNA. The marked reduction was observed in the capped transcript of *TUB1* and *ACT1* genes after 2 h compared to 1 h of inhibitor treatment or untreated parasites (Fig. 7C). The decrement in 5′cappped transcripts following Cdk7 inhibitor treatment further supports the role of Cdk7 in *de novo* mRNA synthesis in *Toxoplasma gondii*.

## Discussion

CDKs and their partners play a central role in coordination of cell cycle and regulation of transcription in metazoans. However, in parasitic protozoans full functional study of such key molecules remained unattended up until now. Here we have shown that Apicomplexan parasite *Toxoplasma gondii* encodes Cdk7 and in possible conjunction with proteins CyclinH and Mat1 phosphorylates Ser5 residue of TgRPB1-CTD. Moreover, we have demonstrated that the inhibitor BS-181 specifically abrogates Cdk7 mediated CTD kinase activity which results into marked reduction of Ser5 phosphorylation, affecting key downstream events including TgRPB1 P-Ser5 occupancy at gene promoters, nascent RNA synthesis and a concomitant decline of capped transcripts. In conclusion, this study demonstrates the role of TgCdk7 in RPB1-driven mRNA synthesis in the parasite *T. gondii*.

The ability of *Toxoplasma* Cdk7, CyclinH and Mat1 to complement the loss of Kin28, Ccl1 and Tfb3 in *S*. *cerevisiae* (Fig. 1B), indicates the possible existence of a complex which may be a part of the general transcription factor like its yeast counter parts^42^. This is consistent with biochemical studies which demonstrated that recombinant TgCdk7 showed maximal TgRPB1-CTD kinase activity *in vitro*, when it is in a complex with TgCyclinH and TgMat1 (Fig. 4B). This observation is in agreement with the mammalian counterparts where the binding of Mat1 to the Cdk7-CyclinH complex stimulates its CTD kinase activity^50^, in addition to stabilization of the TgCdk7-TgCyclinH interaction resulting in an active kinase complex possibly mirroring mammalian CAK^9^,^11^,^12^,^50^. We would like to point out here that we failed to observe any autophosphorylation for TgCdk7 which displayed kinase activity upon cyclin binding in the *in vitro* assays even in absence of TgMat1. This may be explained by the observations, that unlike mammalian system, there is lack of strict cyclin-CDK pairings in the apicomplexan parasites^51^ suggesting a unique mode of activation despite general similarities.

Specific antibodies raised against these proteins, confirmed their expression in the parasite (Fig. 2A–C). A complex of TgCdk7-TgCycilnH-TgMat1 was observed in addition to pairwise interactions using recombinant purified proteins pointing to a possibility of complex formation *in vivo*. The possibility of the presence of an active Tg-Cdk7-CyclinH-Mat1 complex in the parasite was further supported by nuclear co-localization of these proteins; with one of them (i.e. TgCyclinH) bearing canonical nuclear localization signal suggesting a likely TgCyclinH mediated nuclear transport of the associated proteins. Higher resolution imaging and co-IP experiments may provide more robust evidence in favour of the complex formation by these proteins. In this respect, we did attempt to capture the complex from the parasite using co-IP with specific antibodies but failed to detect the same. *In vitro* pull down assays suggested that it may be possible to demonstrate the complex formation with the aid of the tag at the protein termini. Following that cue, we created transgenic parasite lines with YFP or mCherry fusion proteins. This strategy helped us to confirm one of the three interactions (TgCdk7-YFP and TgMat1) while other interactions were still not detectable. The inability to unequivocally validate the interactions in the parasite may be attributed to the fact that the interactions are too transient to capture by conventional methods or may be affected by steric hindrances due to antibodies or immobilizing supports used in the IP. This has also been pointed out in previous studies^37^,^52^,^53^,^54^.

We observed that TgCdk2 is not a substrate of the Tg-Cdk7/CyclinH/Mat1 complex (Fig. 4E) and therefore raised the question as to whether TgCdk7 functions as CAK in *Toxoplasma*. While this observation is consistent with *Plasmodium* Pfmrk (Cdk7 homolog) kinase, which upon activation by PfCy-1 and PfMat1 could not phosphorylate PfPK5^35^,^36^ (Cdk1 homolog), the possibility of a different CDK substrate requirement for this complex may not be ruled out. The ternary complex (Cdk7-CyclinH-Mat1) appears to be conserved throughout eukaryotes however they have evolved to acquire specialized functions in several different organisms. In mammalian cells the Cdk7 complex has a dual role as both CTD kinase and as a CAK. In *S*. *cerevisiae*, the Cdk7 homolog, Kin28 is a CTD kinase but lacks CAK activity. In this respect, TgCdk7 may be more like Kin28 than mammalian Cdk7.

Our study clearly highlights the CTD kinase activity of TgCdk7 as one of its key functions. The CTD of RNA polymerase II consists of multiple heptapeptide repeats (consensus Y_1_S_2_P_3_T_4_S_5_P_6_S_7_), which appears to increase in number with organism complexity^16^. This prompted us to closely look at TgRPB1-CTD and we found that it consists of nine putative heptapeptide, YSPxSPx sequences (Fig. 5A). Examination of the CTD in a variety of organisms revealed a mixture of sequences ordered into multiples of the YSPxSPx based around repeats of the classical, heptapeptide YSPTSPS.

This heptapeptide which is the functional unit of the CTD in a variety of organisms is subject to extensive phosphorylation, during the transcription cycle. Serine 5 and serine 2 phosphorylation of CTD are the most conserved general marks of transcription^16^. CTD is phosphorylated on Ser5 by Cdk7 based trimeric complex CAK near 5′end of genes^17^ whereas phosphorylation of Ser2 is undertaken by Cdk9/cyclin T1, which accumulates as it progress through the gene^18^,^19^. We have shown that *Toxoplasma* Cdk7 phosphorylates both recombinant and endogenous TgRPB1-CTD on Ser5 residue. The key component of positive transcription factor b *i.e.* Cdk9 (TGME49_281450) has been found in *T*. *gondii* database, along with the conserved serine at position 2 of TgRPB1-CTD are critical for transcription elongation in the metazoans^17^,^21^. A cyclin like protein has also been identified (TGME49_264690) which may partner Cdk9 in accomplishing the elongation function. This protein which shares features of two different mammalian cyclins namely CyclinL1 (E value 1e-41) and CyclinT1 (E value 6e-19) may serve other CDKs as well. It has been pointed out before that CDK-cyclin pairings may be flexible in these apicomplexan parasites unlike its mammalian counterparts, due to the limited repertoire of cyclins available^51^. It is now important to investigate whether Cdk9 along with cyclin partner form a complex which is able to phosphorylate CTD on Ser2 for RPB1 mediated productive elongation in the *Toxoplasma*.

Our observations of TgCdk7 inhibition upon BS-181 treatment are mostly in agreement with previous finding^44^. Cdk7 inhibitor, BS-181 protrudes its 3-isopropyl side chain into a kinase pocket formed by the important gatekeeper residues Phe_91_ and the C4 carbon chain of Lys_41_ and thereby inhibits Cdk7 mediated CTD phosphorylation of RNAPII^44^. The gatekeeper residue of protein kinases contributes to the hydrophobic spine of the kinase domain and plays an important role in promoting enzymatic activity. Like mammalian Cdk7, TgCdk7′s kinase activity was markedly reduced upon BS-181 inhibitor treatment. In *Toxoplasma* Cdk7 where critical gatekeeper residues Phe and Lys are conserved, similar inhibitory mechanism may be at play.

We demonstrate that phosphorylation by Tg-Cdk7/CyclinH/Mat1 kinase complex on Ser5 results in a TgRPB1-CTD that can no longer bind DNA (Fig. 6). This dissociation from DNA may facilitate further phosphorylation of CTD at conserved serine 2 by Cdk9 like kinase. Our results are in close agreement with mammalian Cdk7 mediated CTD phosphorylation studies that arrived at similar conclusions^21^.

TgCdk7 emerges as a critical Ser5 kinase when assayed with chemical inhibitor. Our data showed that the endogenous TgRPB1-CTD Ser5 phosphorylation is essentially abolished at micromolar concentrations of the inhibitor, highlighting the potential importance of CTD phosphorylation in this context. Upon inhibitor treatment TgRPB1-CTD P-Ser5 occupancy was decreased on previously characterized gene promoters, *TUB1* and *SAG1*^45^,^46^ (Fig. 7A). Additionally, inhibitor treatment caused decrease in nascent RNA synthesis of two highly expressed *Toxoplasma* genes, *TUB1* and *ACT1* (Fig. 7B). Although strong changes were measured on *TUB1* and *ACT1* genes further studies are warranted to check the global effect in transcription upon Cdk7 inhibition.

Eukaryotic pre-mRNAs are transcribed by RNA polymerase II and undergo several co-transcriptional processing events such as 5′end capping, intron removal by splicing, and 3′end formation before maturing into mRNA. The pre-mRNA capping occurs after only 20 to 30 nucleotides have been transcribed and is the first detectable mRNA processing event^16^. Capping is restricted to Pol II transcripts by capping enzyme recruitment to a phosphorylated CTD. This interaction is mediated by a direct association of the capping enzyme with the phosphorylated CTD^29^. We demonstrated that the inhibition of TgCdk7 kinase activity leads to marked reduction in the gene specific capped transcripts. Thus, the role of TgCdk7 as CTD kinase is probably important for 5′ capping of transcripts and for downstream events that associate with TgRPB1 during different stages of transcription. This is consistent with studies of the mammalian capping enzyme which binds to a CTD phosphorylated at serine 5^17^. Therefore, TgRPB1 promoter clearance/dissociation, reduction in nascent RNA and a concomitant decline of capped transcript are observed in response to TgCdk7 inhibition and are consistent with the widely held view that CTD phosphorylation by Cdk7 is essential for mRNA synthesis^55^.

We demonstrate for the first time that a Cdk7 kinase activity is key for Pol II-driven transcription in the protozoan parasite *Toxoplasma gondii* which confirm with the widely accepted models for the role of Cdk7 kinase activity in transcription initiation and elongation. This study gives clear insights into the processes of nascent RNA synthesis and 5′ capping using approaches not previously used for parasites. Now it is open for further investigation whether TgCdk7 has atypical, parasite specific functions, and its potential as a target for disruption of overall parasite progression. Generation of TgCdk7 knockout or conditional knockout may allow a more precise analysis of its role in the unique aspects of the *Toxoplasma* cell cycle and transcription.

## Methods

The animal experiments conducted were approved by the NIAB Institutional Animal Ethics Committee (IAEC, Reference No. TBPL-NIAB/01/2014). All experimental protocols followed were in accordance with the guidelines approved by NIAB Institutional Biosafety committee (IBSC, Reference No. IBSC/2014/NIAB/004).

### Parasite culture

*T*. *gondii* RH strain was propagated *in vitro* in Human foreskin fibroblasts (H27, ATCC CRL1634) cells grown in Dulbecco modified Eagle medium supplemented with 10% fetal calf serum, 10 mM HEPES (pH 7.4), 1 mM glutamine and 10 μg of gentamycin/ml under 5% CO_2_ at 37 °C. Parasites were harvested by filtration through 3.0-μM-Nucleopore filters and collected by centrifugation at 400 × g for 10 min as described^56^.

### DNA manipulation

Sequences encoding ORF of full length TgCdk7_1-425_, TgCyclinH_1-600_, TgMat1_1-279_, TgCdk2_1-300_ and carboxyl-terminal domain of TgRPB1_1570-1828_ (subscript numbers denote amino acid coordinates) were amplified by PCR using *T*. *gondii* cDNA and specific primers (Supplementary Table S1) and cloned into pGEX-6P-2, pGEX-6P-2, pET-28a, pET-21c and pET-28a vector between *BamH*I-*Xho*I, *BamH*I-*Sal*I, *Nde*I-*BamH*I, *NdeI*-*Xho*I and *BamH*I-*EcoR*I sites respectively. For pull down experiments, TgCdk7 was cloned into pET-28a vector whereas TgMat1 was cloned into pGEX-6P-2 and pMALc5X vectors. The resulting recombinant clones were sequenced.

### Complementation of yeast mutants

For complementation assays, *ScKin28*, *ScCcl1* and *ScTfb3* chromosomal copy deletion mutant stains carrying the wild-type copy in a plasmid with *URA* marker were used^42^. Full-length *T. gondii* genes (*Cdk7*, *CyclinH* and *Mat1*) and *S*. *cerevisiae* genes (*Kin28*, *Ccl1* and *Tfb3*) were amplified by PCR using either *T*. *gondii* or *S*. *cerevisiae* cDNA and specific primers (Supplementary Table S1). This was followed by cloning them in pYES3/CT yeast expression vector, which has tryptophan selection marker and C-terminal His-tag. All constructs were confirmed by sequencing. Yeast mutant strains were transformed with empty vector (pYES3/CT) or vector carrying *T*. *gondii* or *S*. *cerevisiae* gene of interest and grown on synthetic defined (SD) medium as described^57^. For selection yeast strains were streaked onto SD –Trp plates with or without 5-Fluoroorotic Acid (5-FOA). Expression of TgCdk7, TgCyclinH, and TgMat1 in the yeast mutant strains were detected by anti-His antibody.

### Purification of recombinant proteins

His and GST-tagged recombinant proteins were purified as described previously^58^. The following proteins were used in the study: His-TgCdk7, His-TgMat1, TgCdk2-His, His-TgRPB1-CTD, GST-TgCdk7, GST-TgCyclinH, GST-TgMat1, MBP-TgMat1 and histone H1 (M2501S, New England Biolabs). His-TgMat1 was purified from the inclusion bodies.

### Antibodies and reagents

Polyclonal antibodies against TgCdk7, TgCyclinH, TgMat1 and TgRPB1 were raised in mice using following purified GST-TgCdk7, GST-TgCyclinH, His-TgMat1 and His-TgRPB1_1570-1828_ (CTD) proteins respectively. For colocalization studies, polyclonal antibody against TgCyclinH was raised in rabbit. The following commercial antibodies were used: His (H1029, Sigma), anti-GFP antibody (ab6556, Abcam), anti-mCherry antibody (PA5-34974, Thermo Scientific), anti-RNAPII CTD repeat P-Ser5 (ab5131, Abcam), anti-RNAPII CTD repeat P-Ser2 (ab5095, Abcam), control IgG (ab46540; Abcam) and anti-m3G-cap &m7G-cap (MABE419, Millipore), anti-MBP antibody (E8032S, New England Biolabs).

### Fluorescence microscopy

An HFF monolayer was grown on coverslips in a 6-well plate and infected with either RH or TgCdk7-YFP or TgMat1-mCherry or TgCyclinH-YFP parasites. At 16 to 24 h post infection, the infected cell monolayer was fixed in 4% paraformaldehyde. For immunofluorescence analysis intracellular parasites were stained as previously described^59^. The primary polyclonal antibodies against TgCdk7, TgCyclinH, TgMat1 and TgRPB1 were used at dilution 1:200. All Alexa-conjugated secondary antibodies (Alexa Fluor-488 or Alexa Fluor-594) were used at dilution 1:1000. The coverslips were mounted with Antifade containing DAPI (4′,6-diamidino-2-phenylindole) (P36931, ThermoFisher Scientific) on glass slide and viewed on Leica Confocal microscope with 100× objective. Images were collected and processed using LAS X software.

### Yeast two-hybrid analyses

All yeast reagents and methods utilized for yeast-two-hybrid assays were as per the Matchmaker Gold Yeast Two-Hybrid System (630489, Clontech). Bait plasmids were constructed by cloning full length ORF of CyclinH and Mat1 into the pGBKT7 vector whereas pray plasmids were constructed by cloning full length ORF of Cdk7, CyclinH and Cdk2 into the pGADT7 vector. To check the interaction between *T*. *gondii* proteins, respective bait and pray plasmids were cotransformed into *S*. *cerevisiae* strain Y2HGold using the lithium acetate method and plated onto SD plates lacking Leu and Trp. The colonies were subsequently transfer to medium lacking His, Leu and Trp supplemented with 10 mM 3-amino-triazole and for higher stringency onto SD plates lacking Ade, His, Leu and Trp for growth selection. For spot assays, serial dilutions were prepared (OD_600_) as 1, 0.1, 0.001, 0.001 and 0.0001 and plated onto SD/-Leu/-Trp, SD/-His/-Leu/-Trp and SD/-Ade-His/-Leu/-Trp plates.

### Pull-down assay

3 μg of GST or GST-TgCyclinH or GST-TgMat1 bound on Glutathione-agarose beads were incubated with 1 μg of soluble His-TgCdk7 or His-TgMat1 or Cdk2-His for 1 h in 20 mM Tris pH 7.5, 0.2 M NaCl, 0.1% Nonidet P40 and 10% glycerol at 4 °C. The tubes were rotated on a nutator at 4 °C for 1 h. After centrifugation, the beads were recovered and washed four times in the same buffer, resuspended in Laemmli buffer, boiled and loaded on 12% SDS-PAGE to analyse the bound protein by Western blot using respective antibodies.

For *in vitro* interaction studies, co-immunoprecipitation (Co-IP) reactions were performed as described^37^. Briefly, 2 μg of each protein in the reaction was allowed to incubate on ice for an hour with frequent mixing. The proteins were incubated with anti-MBP antibody crosslinked to aminoLink agarose (Co-IP kit, 26149, Pierce) at 4 °C on a rotary shaker for 1 h. The beads were washed with Co-IP buffer followed by elution of bound proteins. The eluted proteins were boiled with 2x SDS-PAGE gel loading buffer and subjected to silver staining.

The IP from parasite proteins was performed using co-IP IP kit (26149, Pierce) as described by manufacturer. Briefly, parasites (10 × 10^8^ ml^−1^) were lysed in the lysis buffer provided in the kit in the presence of a protease and phosphatase inhibitor cocktails. Protein normalized parasite lysates were immunoprecipitated using appropriate antibody crosslinked to aminoLink agarose at 4 °C, overnight. The immunoprecipitates were recovered after centrifugation, and the eluted proteins were subjected to SDS-PAGE followed by Western blot analysis.

### Kinase assay

Kinase assays were performed as described^60^. Briefly, 1.0 μg of TgRPB1-CTD or histone H1 proteins were assayed at 30 °C for 30 min in a 30 μl kinase reaction buffer supplemented with 3 μg of various combinations of Tg-Cdk7/CyclinH/Mat1/Cdk2 proteins or parasite lysate in absence or presence of BS-181 (Sc-364448, Santa Cruz) (10 nM–40 nM). Following the incubation, the reactions were resolved on a 12% SDS-PAGE gels and later visualized by phosphorimager.

Preparation of parasite lysate for *in vitro* kinase assay: lysate was prepared from parasites treated with DMSO or BS-181 (5 μM and 10 μM) for 6 h using extraction buffer essentially described previously^60^. The concentrations of extracted proteins were measured by Bradford assay. Kinase reactions were performed using 10 μg total protein lysate.

### Immunodepletion

Parasites were lysed at 4 °C by placing them in a rotating platform in the presence of lysis buffer (50 mM Tris-HCl pH 7.5, 1% Triton X-100, 1 mM DTT, 50 mM Na_3_VO_4_, 50 mM NaF, 50 mM β-glycerophosphate, protease and phosphatase inhibitor cocktail). Centrifuge separated supernatant was incubated with TgCdk7 antiserum or pre-immune antibody crosslinked to aminoLink agarose on a nutator at 4 °C for 6 h. The beads were separated by centrifugation and supernatants were used for kinase assay.

### Electrophoretic mobility shift assay (EMSA)

EMSA was carried out using radiolabeled *T*. *gondii* Tubulin promoter DNA (~140 bp) amplified from parasite expression vector sagCATsag_Tub2358YFP (Supplementary Fig. S9) using the primers (Supplementary Table S1). DNA binding reactions were performed in presence of unphosphorylated or phosphorylated His-CTD as described^58^. Kinase reaction was performed by incubating TgRPB1-CTD with different concentrations of bead bound Tg-Cdk7/CyclinH/Mat1 in the presence or absence of inhibitor, BS-181 for 45 min at 30 °C prior to EMSA reaction. Gels were visualized by phosphorimager.

### Chromatin immunoprecipitation (ChIP)

ChIP was performed as described^61^ with the following modifications. Chromatin from intracellular tachyzoites grown in HFF for 24 h was cross-linked at room temperature for 10 min with 1% formaldehyde. The cross-linked DNA was sheared to average size of 500 bp and was immunoprecipitated using respective antibody at 4 °C overnight. Thirty microliters of 50–50 slurry of Protein G agarose bead was added to the IP and tubes were nutated at 4 °C for an additional hour. DNA was further subjected to a treatment with proteinase K for 2 h and then purified using phenol:chloroform. The control IgG antibody was used as a negative control. Immunoprecipitated DNA from inhibitor treated parasites was quantified and normalized by calculating fold enrichment over nontranscribed telomere DNA^62^.

### RT-qPCR analysis

Total RNAs from control or BS-181 treated parasites were isolated by TRIzol (15596-026, Invitrogen). DNase I treated RNAs were used to generate cDNA using random hexamers and SuperScipt III reverse transcriptase (12574018, Invitrogen) following the manufacturer’s protocol. Real-time quantitative PCR was performed on the 7500 ABI apparatus using cDNA samples in conjunction with SYBR green PCR Master Mix (ABI). The nascent RNA levels were determined as described previously^47^. Briefly, the nascent RNA levels of *TUB1* and *ACT1* were determined using primers targeting specific exon-intron junctions, which allowed quantitative analysis of newly synthesized pre-mRNA. Amplification from exonic region of pre-existing (basal) mRNA of the Actin which is expected to be unaltered was used as controls.

Three replicate reactions were performed for each sample using the following cycle conditions: 95 °C, 15 min followed by 40 cycles of 94 °C, 30 s; 55 °C, 40 s and 68 °C, 50 s. Relative transcript levels were calculated by the ∆∆*C*_*T*_ (where *C*_*T*_ is threshold cycle) method.

### RNA ChIP of 5′-capped transcript

RNA immunoprecipitation was performed as described^63^ with some modifications. ChIP was performed as described earlier from DMSO and BS-181 treated parasites using anti-cap (anti-m3G-cap & m7G-cap) antibody and control IgG antibody. Immunoprecipitated RNA was recovered by phenol:chloroform extraction followed by ethanol precipitation. The RNA pellets were resuspended in DEPC-treated water. The cDNA was prepared using random hexamers and reverse transcriptase as described in RT-PCR method. Real-time PCR was performed using specific primers of (Supplementary Table S1) *TUB1* and *ACT1*. RT-qPCR amplifications from exonic region of pre-existing mRNA of Actin gene was used as control for quantification.

### *T*. *gondii* transfection

To generate *T*. *gondii* transgenic parasites stably expressing exogenous copies of either TgCdk7 or TgMat1 or TgCyclinH, plasmids were constructed based on *T*. *gondii* expression vectors: sagCATsag_Tub2358YFP and sagCATsag_Tub2358mCherry^56^ (Kind gift from Dr. Dhanasekaran Shanmugam, NCL, Pune, India). Both the vectors facilitate the expression of recombinant proteins in *T*. *gondii* driven by the tubulin promoter, with a C-terminal yellow fluorescent protein (YFP) fusion or mCherry fusion. Plasmids were constructed by removing an insert with *Bgl*II/*Avr*II sites from the original vector and cloning the respective *T*. *gondii* gene in the same sites. Transfections in *T*. *gondii* RH strain were performed as described previously^56^ using Bio-Rad X-cell Electroporator (settings of 1500 V, 25 μF and square wave). Tachyzoites were transfected with 100 μg of TgCdk7-YFP or TgCyclin-YFP or TgMat1-mCherry plasmid to generate single expression strain. The transfected parasites were inoculated on HFF monolayer grown on coverslips or T25 flasks and selected with 20 μM chloramphenicol 24 h postinfection. YFP or mCherry fluorescent parasites were clonally selected in a 96-well plate by limiting dilution. Selected single clonal lineage of transgenic parasite were then propagated and upscaled for immunoprecipitation experiments.

### Flow cytometry

Apoptosis analysis of HFF cells treated with BS-181 for 24 h was undertaken by dual labelling of cells with Annexin-fluorescein and propidium iodide using an Annexin V-FITC apoptosis detection kit I (556547, BD Biosciences) followed by flow cytometry measurements.

## Additional Information

**How to cite this article**: Deshmukh, A. S. *et al*. Cdk7 mediates RPB1-driven mRNA synthesis in *Toxoplasma gondii.* *Sci. Rep.* **6**, 35288; doi: 10.1038/srep35288 (2016).

## Supplementary Material

Supplementary Information
