# Supplementary material for: Cdk7 mediates RPB1-driven mRNA synthesis in Toxoplasma gondii
Source: Sci Rep. 2016 Oct 19;6:35288. doi: 10.1038/srep35288 (PMC5069487; doi:10.1038/srep35288)
Supplement: Supplementary Information [file srep35288-s1.pdf]

SUPPLEMETARY INFORMATION

Cdk7 mediates RPB1-driven mRNA synthesis in *Toxoplasma gondii*

Abhijit S. Deshmukh[1]*, Pallabi Mitra[2] and Mulaka Maruthi[3]

[1] National Institute of Animal Biotechnology, Hyderabad, India, [2] Laboratory of Transcription, Centre for DNA Fingerprinting and Diagnostics, Hyderabad, India and [3] Department of Animal Sciences, University of Hyderabad, Hyderabad, India

Supplementary method

Proliferation of *T. gondii*

Parasites inside the parasitophorous vacuoles (PVs) were counted as described previously (**Zhou et al., 2013**). HFF cells were cultured in 24-well plates containing glass coverslips prior to infection with *T. gondii* at a multiplicity of infection (moi) of 5. Parasite synchronization was achieved by washing off free, uninfected parasites after 2 h of infection. After 24 h of BS-181 treatment, the coverslips were washed with PBS and then fixed with 4% paraformaldehyde and scanned under the microscope. One hundred parasitophorous vacuoles (PVs) were randomly selected in each preparation, and parasite replication was monitored by counting the number of tachyzoites per PV. Three separate experiments were performed for statistical analysis of the results.

Reference

Zhou, W., Quan, J. H., Lee, Y. H., Shin, D. W. & Cha, G. H. Proliferation Require Down-Regulation of Host Nox4 Expression via Activation of PI3 Kinase/Akt Signaling Pathway. *PloS one* **8**, e66306, doi:10.1371/journal.pone.0066306 (2013).

Fig. S1

The CLUSTALW2 Multiple Sequence Alignment of conserved domains of *H. sapiens* (Hs), *S. cerevisiae* (Sc), *P. falciparum* (Pf) and *T. gondii* (Tg) CAK homologs (TgCdk7, TgCyclinH and TgMat1). '**.**', '**:**' and '*****' indicate weakly homologous, similar and identical residues respectively.

Percent Identity Matrix - created by Clustal2.1 for full length protein

1:	HsCdk7	100.00	47.19	**40.45**	42.76
2:	ScKin28	47.19	100.00	**36.75**	33.89
3:	TgCDK7	40.45	36.75	**100.00**	45.37
4:	PfMrk	42.76	33.89	**45.37**	100.00

Percent Identity Matrix - created by Clustal2.1 for conserved protein kinase domain

1:	HsCdk7	100.00	49.82	**43.46**	44.24
2:	ScKin28	49.82	100.00	**38.93**	35.51
3:	TgCdk7	43.46	38.93	**100.00**	46.64
4:	PfMrk	44.24	35.51	**46.64**	100.00

```
HsCdk7          YEKLDFLGEGQFATVYKARDKNTNQIVAIKKIKLGHRSEA--------------------
ScKin28         YTKEKKVGEGTYAVVYLGCQHSTGRKIAIKEIK---TSEF--------------------
TgCdk7          RQCDAFLGEGTYGRVEKAEDLRTHQIVAIKKVKASAGSLFASGDSGSVGSGEKTRAQLLR
PfMrk           IFKPNFLGEGSYGKVYKAYDTILKKEVAIKKMKLN----------------------KIS
                      :*** :. *  . :    : :***::*

HsCdk7          ----KDGINRTALREIKLLQELSHPNIIGLLDAFGHKSNISLVFDFMETDLEVIIKDNSL
ScKin28         ----KDGLDMSAIREVKYLQEMQHPNVIELIDIFMAYDNLNLVLEFLPTDLEVVIKDKSI
TgCdk7          QNVGSVGLHFTTVRELKVMREIEEENVMGVVDVFVEQDFICLVMELMHGDLKKLVDSK-T
PfMrk           NYIDDCGINFVLLREIKIMKEIKHKNIMSALDLYCEKDYINLVMEIMDYDLSKIINRK-I
                    . *:.   :**:* ::*:.. *::  :* :   . : **::::  **. ::. :

HsCdk7          VLTPSHIKAYMLMTLQGLEYLHQHWILHRDLKPNNLLLDENGVLKLADFGLAKSFGSP--
ScKin28         LFTPADIKAWMLMTLRGVYHCHRNFILHRDLKPNNLLFSPDGQIKVADFGLARAIPAP--
TgCdk7          RLAIQHVKCIMLQILRGLHALHKRYIVHRDLAPANVFINDQGICKVADFGLSRCFGCPVV
PfMrk           FLTDSQKKCILLQILNGLNVLHKYYFMHRDLSPANIFINKKGEVKLADFGLCTKYGYDMY
                 ::  . *. :*  *.*:   *: :::**** * *:::. .*  *:*****.

HsCdk7          -------------------------------------NRAYTHQVVTRWYRAPELLFGARM
ScKin28         -------------------------------------HEILTSNVVTRWYRAPELLFGAKH
TgCdk7          SGTLSKQEQSKGETSQPGKESSVESASKTAPVAISRKELMTSKVVTLWYRPPELLFGADR
PfMrk           SDKLFRDKY-------------------------KKNLNLTSKVVTLWYRAPELLLGSNK
                                                    .   * :*** *** ****:*:

HsCdk7          YGVGVDMWAVGCILAELLLRVPFLPGDSDLDQLTRIFETLGTPTE---------EQWPDM
ScKin28         YTSAIDIWSVGVIFAELMLRIPYLPGQNDVDQMEVTFRALGTPTD---------RDWPEV
TgCdk7          YGQAVDMWSVGCIMAELLTGSPLFPGANEIDQLSRIFSLRGTPTTAAALLDEEPSLWPLA
PfMrk           YNSSIDMWSFGCIFAELLLQKALFPGENEIDQLGKIFFLLGTPNE---------NNWPEA
                *  .:*:*:.* *:***:     :** .::**:   *   ***.            **

HsCdk7          CSLPDYVTFKSFPGIP---LHHIFSAAGDDLLDLIQGLFLFNPCARITATQALKMKYF
ScKin28         SSFMTYNKLQIYPPPSRDELRKRFIAASEYALDFMCGMLTMNPQKRWTAVQCLESDYF
TgCdk7          SSLPSFFPFTHTKPKS---LKSVLPFCCADSLDLLDKLLQLDPSKRITAAEALNHRWF
PfMrk           LCLPLYTEFTKATKKD---FKTYFKIDDDDCIDLLTSFLKLNAHERISAEDAMKHRYF
                 .:  :  :          ::  :       :*::  :: ::   * :* :.::  :*
```

Fig. S1A

Percent Identity Matrix - created by Clustal2.1 for full length protein

		1	2	3	4
1:	PfCyclin1	100.00	**21.81**	19.71	15.87
2:	TgCyclinH	21.81	**100.00**	22.68	20.53
3:	HsCyclinH	19.71	**22.68**	100.00	28.38
4:	ScCcl1	15.87	**20.53**	28.38	100.00

Percent Identity Matrix - created by Clustal2.1 for cylcin box

		1	2	3	4
1:	HsCyclinH	100.00	31.71	**25.93**	29.63
2:	ScCcl1	31.71	100.00	**21.25**	26.58
3:	TgCyclinH	25.93	21.25	**100.00**	34.15
4:	PfCyclin1	29.63	26.58	**34.15**	100.00

```
HsCyclinH       --RLLEFCSVFKPAMPRSVVGTACMYFKRFYLNNSVMEYHPRIIMLTCAFLACKVDEFNV
ScCcl1          YAKKVQ-VIAQHLNLPTEVVATAISFFRRFFLENSVMQIDPKSIVHTTIFLACKSENYFI
TgCyclinH       -FQLVLICKRKR--VKLPVIETACVYLHRFFCMRSPLAFDIRLVIFACLLLALKAEDVAR
PfCyclin1       --QLVHFCEIKM--LRPHIVECATILYNRFYLKEIILEYDPRILIFTCIVLAIKIEGYGR
                  : :         :   ::  *    .**:  .  :  . : :: :  .** * :

HsCyclinH       --SSPQFVGNLRESPLGQEKALEQILEYELLLIQ
ScCcl1          --SVDSFAQKAKSTR-------DSVLKFEFKLLE
TgCyclinH       HYSLGDLLGDIAELDI------GEVLRLELPVC-
PfCyclin1       LYKINEFFNDID-INL------DKVLEHENIVCS
                  .  .:  .             .:*. *  :
```

Fig. S1B

Percent Identity Matrix - created by Clustal2.1 for full length protein

1: TgMat1	**100.00**	33.85	28.69	30.23
2: PfMat1	**33.85**	100.00	24.39	26.21
3: HsMat1	**28.69**	24.39	100.00	32.29
4: ScTfb3	**30.23**	26.21	32.29	100.00

Percent Identity Matrix - created by Clustal2.1 for full Ring finger domain

1: HsMat1	100.00	50.00	**39.02**	29.55
2: ScTfb3	50.00	100.00	**35.71**	26.67
3: TgMat1	39.02	35.71	**100.00**	41.86
4: PfMat1	29.55	26.67	**41.86**	100.00

```
HsMat1      CPRCKTTKYRNPSLKLMVN-VCGHTLCESCVDLLFVRGA------GNCPEC--
ScTfb3      CPICKTDRYLSPDVKFLVNPECYHRICESCVDRIFSLGP------AQCPYKGC
TgMat1      CPVCYESCYFHPERKLFHSDVCKHRICGSCLHIHFGENGARGERRGFCPVCR-
PfMat1      CISCFEDIYVNNEKKLYFFDICKHKICGECLENHLNK-----LNKQYCPLC--
            *  *    *   . *:     * * :* .*:.  :            **
```

Fig. S1C

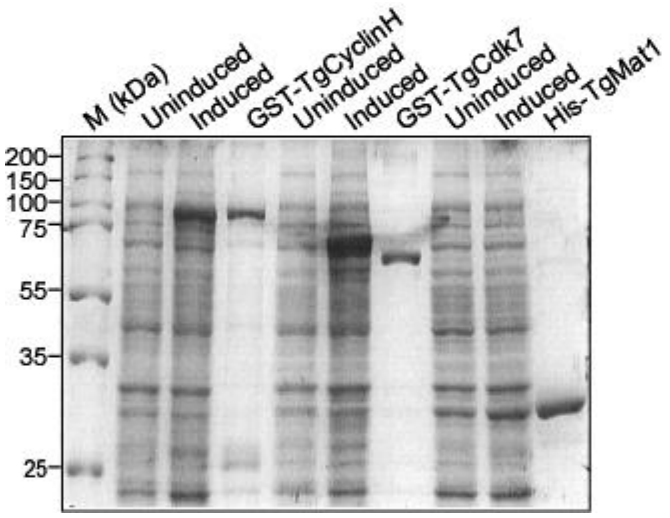

Fig. S2 Recombinant protein expression of full length TgCyclinH, TgCdk7 and TgMat1. Coomassie gel showing uninduced, IPTG induced and purified GST-TgCyclinH (~92kDa), GST-TgCdk7 (~72kDa) and His-TgMat1 (~32kDa) proteins.

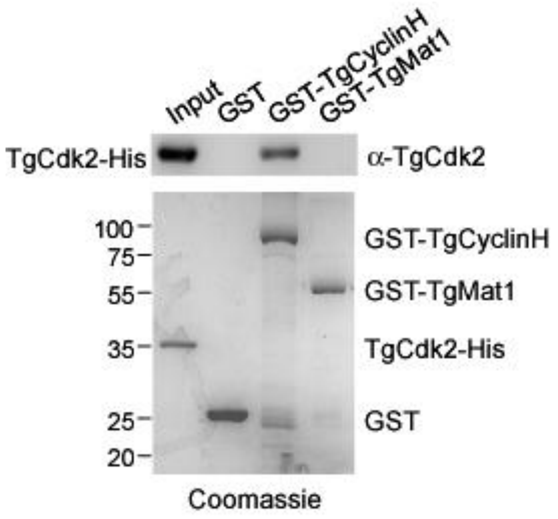

Fig. S3 GST beads bound TgCyclinH, TgMat1 and GST alone proteins in the presence of TgCdk2-His showed the interaction of TgCyclinH with TgCdk2 but no interaction of TgMat1 with TgCdk2 was detected.

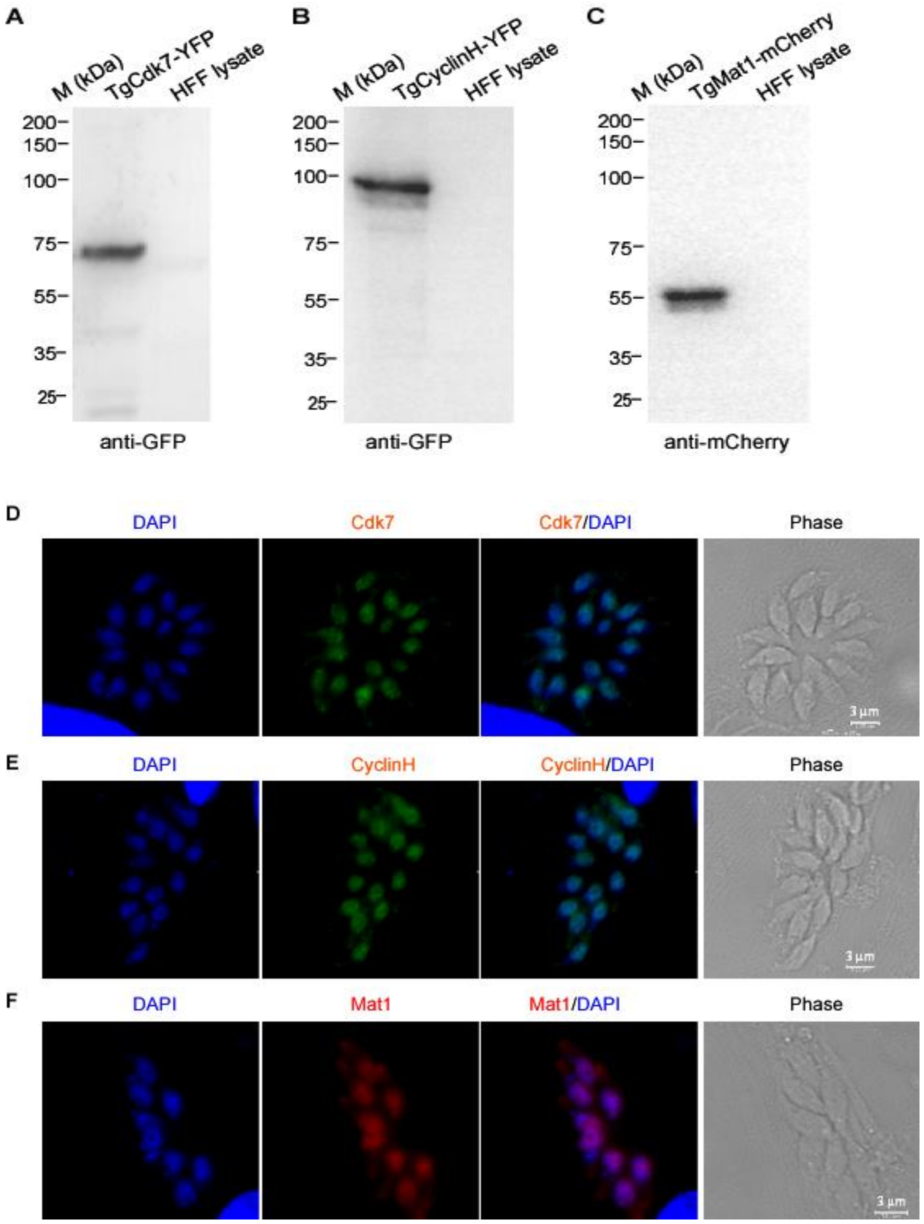

Fig. S4 (**A,B**) Anti-GFP antibody specifically recognized the TgCdk7-YFP and TgCyclinH-YFP fusion protein bands at ~72kDa and ~92kDa sizes respectively. (**C**) Similarly, anti-mCherry antibody specifically recognized the TgMat1-mCherry fusion protein band at ~58kDa size. Both anti-GFP and anti-mCherry antibodies did not display cross-reactivity with uninfected HFF lysate. (**D-F**) All three fusion proteins namely TgCdk7-YFP, TgCyclin-YFP and TgMat1-mCherry showed localization in the parasite nucleus. DAPI was used to stain the parasite nucleus.

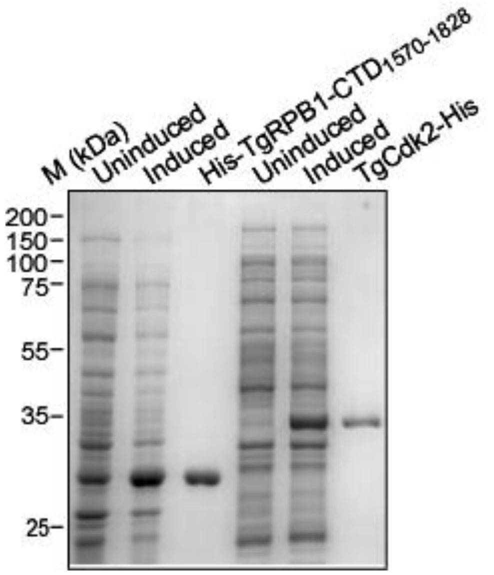

Fig. S5 Recombinant protein expression of TgRPB1-CTD$_{1570-1828}$ and TgCdk2. Coomassie gel showing uninduced, IPTG induced and purified His-TgPOLR2A-CTD (~27kDa) and TgCdk2-His (~35kDa) proteins.

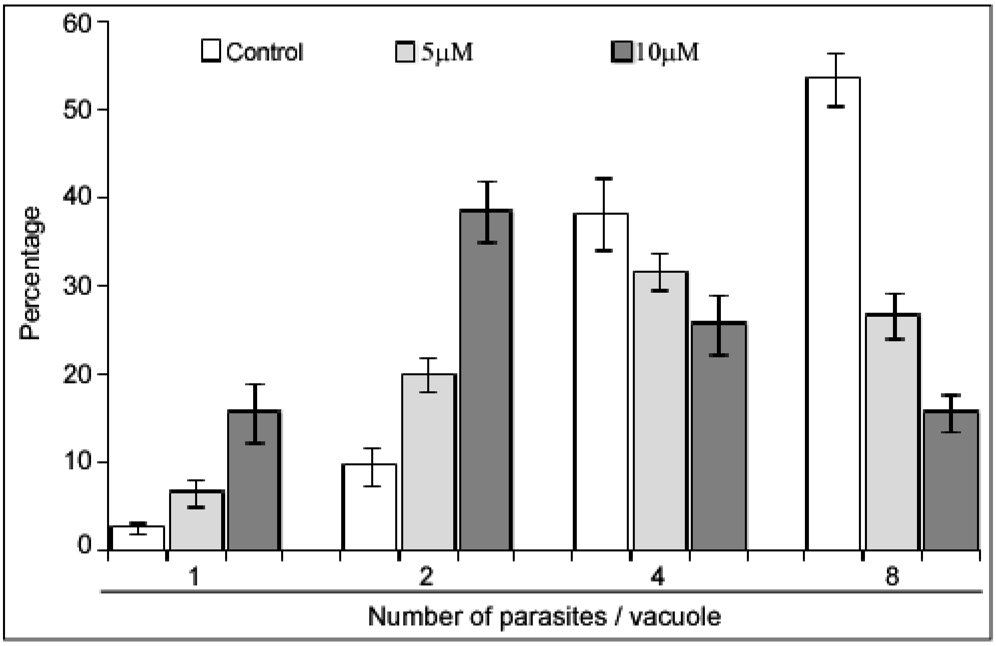

Fig. S6 Cdk7 inhibitor, BS-181 inhibited *T. gondii* proliferation in HFF cells. The proliferation of tachyzoites in HFF cells was examined by microscopy. Cells were infected with *T. gondii* at moi of 5 for 24 h. Cells were fixed and observed under microscope. The number of parasites per vacuole were counted and converted to percentage. Data are represented for three independent experiments.

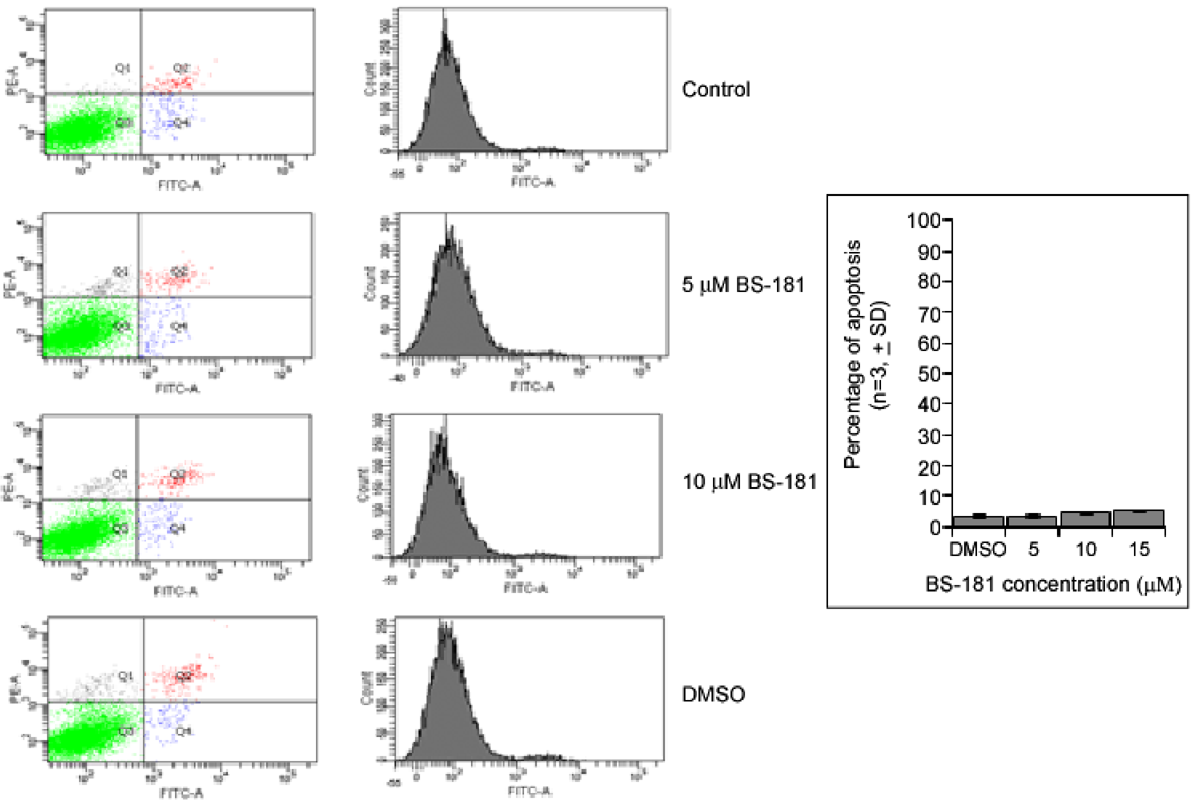

Fig. S7 BS-181 treatment at concentrations of 5 µM and 10 µM do not lead to apoptosis of HFF cells. HFF cells were treated with BS-181 at the concentration of 5 µM or 10 µM or with vehicle (DMSO) for 24 h. Cells were stained with an antibody for Annexin V and with propidium iodide followed by flow cytometric analysis (left panel). The percentage of cells (right panel) that stained positive for Annnexin V are shown for three independent experiments.

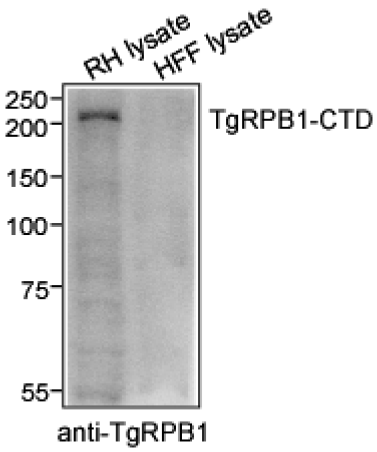

Fig. S8 Generation of specific polyclonal antibodies against TgRPB1. Polyclonal antibodies raised using His-TgRPB1-CTD as antigen were specific as western blot analysis with TgRPB1 antiserum recognized the band of expected size (~205 kDa) in the native parasite lysate while it showed no cross reactivity with uninfected HFF lysate.

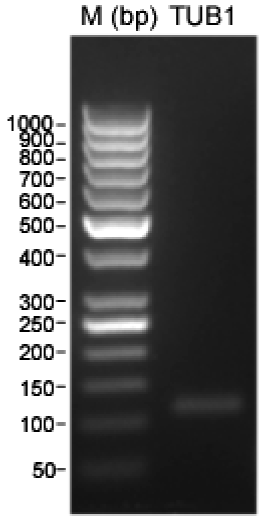

Fig. S9 PCR amplification using primer pairs (Table S1) specific for TUB1 promoter region as described in methods. Standard molecular mass markers (bp) are shown on left. The result indicates single band PCR amplification around 140 bp.

Table S1 List of primers used in this study

Sr. No.	Primer Name	Primer sequence	Accession No.	Plasmid/Use
1	TgCdk7_1BamHIF	5’CGGGATCCATGGCGGCACCGCAAGTC	TGME49_270330	pGex6P2 &YFP
2	TgCdk7_1278XhoIR	5’CCCTCGAGTCAGAATTTGCCGAGAAGATG		
3	TgCDK7_1NdeIF	5’GGAATTCCATATGGCGGCACCGCAAGTC	TGME49_270330	pGADT7
4	TgCDK7_1275XhoIR	5’CCCTCGAGGAATTTGCCGAGAAGATGGA		
5	TgCyclinH_1BamHIF	5’CGGGATCCATGGCTTCTGATTCTCCCG	TGME49_260250	pGex6P2,mCherry
6	TgCyclinH_1803SalIR	5’GCGTCGACCTACGGACGAGACGATGTA		
7	TgCyclinH_1BamHIF	5’CGGGATCCATGGCTTCTGATTCTCCCG	TGME49_260250	pGBKT7, pGADT7
8	TgCyclinH_1800SalIR	5’GCGTCGACCGGACGAGACGATGTAACT		
9	TgMat1_1NdeIF	5’GGAATTCCATATGGATAACTACGACTGTCCG	TGME49_320070	pET21c, pGBKT7
10	TgMat1_840BamHIR	5’TCCCCCGGGCTACGCGGATCTTTGCTTCCCT		
11	TgMat1_1BamHIF	5’CGGGATCCATGGATAACTACGACTGTCCG	TGME49_320070	pGex6P2
12	TgMat1_840SmaIR	5’TCCCCCGGGCTACGCGGATCTTTGCTTCCCT		
13	TgCdk2_1NdeIF	5’GGAATTCCATATGGAGAAGTATCAGAAGCTG	TGME49_218220	pET21c, pGADT7
14	TgCdk2_900XhoIR	5’CCCTCGAGTTCGCCCCTGAAAGTAG		
15	TgPOLR2A_4710BamHIF	5'CGGGATCCCTGGGTGGTAAATTTTCTCC	TGME49_225260	pET28a
16	TgPOLR2A_5679EcoRIR	5'CGGAATTCTCAGTGATGGTGATGATGAT		
17	TgCdk7_1BamHIF	5’CGGGATCCAAAAAAAATGTCTGCGGCACCGCAAGTC	TGME49_270330	pYES3/CT
18	TgCdk7_1278XhoIR	5’CCCTCGAGTCAGAATTTGCCGAGAAGATG		
19	TgMat1_1BamHIR	5'CGGGATCCAAAAAAAATGTCTGATAACTACGACTGTCC	TGME49_320070	pYES3/CT
20	TgMat1_837EcoRIR	5’CGGAATTCCGCGGATCTTTGCTTCCCTT		
21	TgCyclinH_1BamHIR	5'CGGGATCCAAAAAAAATGTCTGCTTCTGATTCTCCC	TGME49_260250	pYES3/CT
22	TgCyclinH_1800SalIR	5’GCGTCGACCGGACGAGACGATGTAACT		
23	ScKin28_1HindIIF	5’CCCAAGCTTAAAAAAATGAAGGTGAACATGGAGTAC	P06242	pYES3/CT
24	ScKin28_918XhoIR	5’CCGCTCGAGGTTCCTGATCTTGATGCTGC		
25	ScCcl1_1HindIIF	5’CCCAAGCTTAAAAAAATGACCGACATCCAGCTGAA	P37366	pYES3/CT
26	ScCcl1_1179XhoIR	5’CCGCTCGAGGGTCTTCTGCTTCTTCTCCA		
27	ScTfb3_1HindIIF	5’CCCAAGCTTAAAAAAATGCTGATGGACGAGTACG	A0A0C5IFR2	pYES3/CT
28	ScTfb3_963XhoIR	5’CCGCTCGAGCAGCTCCTCGCTGATCACG		
29	TgαTUB_5272314ChrXIF	5’TGCGGTTGGTGATCCTGG	TGME49_316400	EMSA, ChIP
30	TgαTUB_5272363ChrXIR	5’TAACACATCTAAAAGTTCACAG		
31	TgSAG1_2664154ChrVIIIF	5’GCCTTTGGCTCCTGAGACG	TGME49_233460	ChIP
32	TgSAG1_2664333ChrVIIIR	5’AGGTGCACATTGTGCGGC		
33	TgαTUB_681F	5’CGCTGGCAAGCACGTGAGTA	TGME49_316400	qRT-PCR
34	TgαTUB_834R	5’ GGGGCACCTGAGTCAGCG		
35	TgACT1_996F	5’TTGTCAAGCACTGCTGTTTGG	TGME49_209030	qRT-PCR
36	TgACT1_1138R	5’CGCTAGAGTCCTCAGCCGC		
37	TgACT1_515F	5’GCCTCACGCCATCATGCGTT	TGME49_209030	qRT-PCR
38	TgACT1_1138R	5’CGCTAGAGTCCTCAGCCGC		
39	TgCyc1_1800AvrIIR	5’TGGA*CCTAGG*CGGACGAGACGATGTAACT	TGME49_260250	mCherry
40	TgCDK_1275AvrIIR	5’TGGA*CCTAGG*GAATTTGCCGAGAAGATGGA	TGME49_270330	YFP
41	TgMAT1_1BglIIF	5’GA*AGATCT*ATGGATAACTACGACTGTCC	TGME49_320070	YFP
42	TgMAT1_837AvrIIR	5’TGGA*CCTAGG*CGCGGATCTTTGCTTCCC		YFP

Note

1. In case of gene cloning primers, number before restriction site denotes nucleotide coordinate in the gene ORF (excluding intron).
2. Restriction site sequence is underlined.
3. In case of ChIP primers, number denotes nucleotide coordinate on the particular chromosome.
4. In case of qRT-PCR primers, number denotes nucleotide coordinate in the gene (including intron).
